# Effects of ambient temperature on influenza-like illness: A multicity analysis in Shandong Province, China, 2014–2017

**DOI:** 10.3389/fpubh.2022.1095436

**Published:** 2023-01-09

**Authors:** Jia Yin, Ti Liu, Fang Tang, Dongzhen Chen, Lin Sun, Shaoxia Song, Shengyang Zhang, Julong Wu, Zhong Li, Weijia Xing, Xianjun Wang, Guoyong Ding

**Affiliations:** ^1^Department of Epidemiology, School of Public Health, Shandong First Medical University and Shandong Academy of Medical Sciences, Jinan, Shandong, China; ^2^Center for Big Data Research in Health and Medicine, The First Affiliated Hospital of Shandong First Medical University and Shandong Provincial Qianfoshan Hospital, Jinan, Shandong, China; ^3^Institute for Communicable Disease Control and Prevention, Shandong Center for Disease Control and Prevention, Jinan, Shandong, China; ^4^Institute of Viral Disease Control and Prevention, Liaocheng Center for Disease Control and Prevention, Liaocheng, Shandong, China

**Keywords:** ambient temperature, influenza-like illness, distributed lag non-linear model (DLNM), multivariate meta-analysis, two-stage analytical method

## Abstract

**Background:**

The associations between ambient temperature and influenza-like illness (ILI) have been investigated in previous studies. However, they have inconsistent results. The purpose of this study was to estimate the effect of ambient temperature on ILI in Shandong Province, China.

**Methods:**

Weekly ILI surveillance and meteorological data over 2014–2017 of the Shandong Province were collected from the Shandong Center for Disease Control and Prevention and the China Meteorological Data Service Center, respectively. A distributed lag non-linear model was adopted to estimate the city-specific temperature–ILI relationships, which were used to pool the regional-level and provincial-level estimates through a multivariate meta-analysis.

**Results:**

There were 911,743 ILI cases reported in the study area between 2014 and 2017. The risk of ILI increased with decreasing weekly ambient temperature at the provincial level, and the effect was statistically significant when the temperature was <-1.5°C (RR = 1.24, 95% CI: 1.00–1.54). We found that the relationship between temperature and ILI showed an L-shaped curve at the regional level, except for Southern Shandong (S-shaped). The risk of ILI was influenced by cold, with significant lags from 2.5 to 3 weeks, and no significant effect of heat on ILI was found.

**Conclusion:**

Our findings confirm that low temperatures significantly increased the risk of ILI in the study area. In addition, the cold effect of ambient temperature may cause more risk of ILI than the hot effect. The findings have significant implications for developing strategies to control ILI and respond to climate change.

## 1. Introduction

At present, the impact of human activities aggravates climatic and environmental changes and the global climate anomaly is becoming more and more obvious. Climate change has become one of the biggest health threats of the twenty-first century (1). A changing climate has drawn wide concerns and caused significant impacts on public health. There may be an increase in mortality and morbidity from influenza-like illness (ILI) due to climatic factors, especially ambient temperature (2–5). ILI is an acute respiratory syndrome with fever and at least one respiratory symptom (cough and/or sore throat) (6). While influenza viruses are the main cause of ILI cases (7, 8), other common causes of ILI include rhinoviruses, respiratory syncytial virus, adenovirus, parainfluenza viruses, and some bacterial infections (8). The annual epidemics of influenza cause 3–5 million cases of severe illness and about 0.29–0.65 million respiratory deaths worldwide, which is a global public concern (9). Evaluating the risk factors for trends of ILI is important for preventing and treating influenza.

Ambient temperature plays an important role in the spread of ILI. Winter and spring are known to be the peak influenza seasons in temperate regions, as well as the season of influenza recurrence (10). Some studies found that the meteorological effects on host susceptibility (11) and viral survival (12) and the influence of social contact on ILI transmission (13) encourage the spread of ILI or influenza. In recent years, extensive environmental epidemiology studies have been conducted to quantify the relationship between ambient temperature and ILI/influenza (6, 14–20). There is, however, a discrepancy among the results of the association between ambient temperature and ILI/influenza. For example, one study in the Jiangsu Province of China showed that the association between temperature and the incidence of ILI presented an approximate “M” shape on the province-wide scale (6). While the association between temperature and influenza showed an approximate “S” shape in Wuhan (14), it showed an “L” shape in Guangzhou (15), and an “N” shape among 30 cities in China (20). In addition, most previous studies were focused on the association between ambient temperature and influenza, but few studies have been focused on the association between ambient temperature and ILI (3, 6, 21), especially in a multicity setting at the provincial level through multivariate meta-analysis. China needs to consider the heterogeneity of ambient temperature and ILI associations among different regions or provinces. A multicity study using the distributed lag modeling strategy with multivariate meta-analysis is a possibly better choice to gain a deeper understanding of ambient temperature on the risk of ILI and support region-specific interventions.

In this study, a distributed lag non-linear model (DLNM) was used to assess the effect of ambient temperature on ILI in specific cities in Shandong Province, China. In addition, a two-stage analysis was conducted to fit the overall effect, heat effect, and cold effect of ambient temperature on ILI in 17 cities of the Shandong Province from 2014 to 2017.

## 2. Materials and methods

### 2.1. Study area

Shandong Province is located on the east coast of China. It includes 16 prefecture-level cities, with a total land area of 157,900 km^2^ and a permanent resident population of 101.5 million at the end of 2021. It belongs to the warm temperate monsoon climate zone, characterized by clear-cut seasonal changes with an annual average temperature of 11–14°C. Laiwu, a prefecture-level city before its incorporation into Jinan, was studied during our study period of 2014–2017 despite its abolishment in 2018. The 17 cities were divided into four regions based on their geographical location and the level of economic development: Central Shandong (Jinan, Taian, Laiwu, Zibo, and Weifang), Jiaodong Peninsula (Qingdao, Yantai, and Weihai), North Shandong (Liaocheng, Dezhou, Binzhou, and Dongying), and South Shandong (Heze, Jining, Zaozhuang, Linyi, and Rizhao). The geographical location of the study area is shown in Supplementary Figure 1.

### 2.2. Data collection

The weekly ILI data from January 2014 to April 2017 were obtained from the China Influenza Surveillance Information System (CISIS). In our study, patients were considered to have ILI if they had acute respiratory infection with fever and at least one respiratory symptom (cough and/or sore throat), according to the technical guidelines for national influenza surveillance. In China, ILI cases are automatically recognized by the hospital information system (HIS) and reported weekly through CISIS by the sentinel hospitals. Information on ILI data included sentinel hospitals, weekly confirmed cases of ILI, ages of ILI patients, and weekly total visiting number of outpatients and emergencies. All data from each sentinel hospital were summarized by Excel as time series and after cleaning the data, the final weekly incidence data for each prefecture-level city was obtained to calculate the weekly visits for incidences of ILI.

Daily meteorological data from 2014 to 2017 were obtained from the China Meteorological Data Service Center (http://data.cma.cn/). The meteorological variables included daily average ambient temperature, daily average relative humidity, daily average wind speed, and daily precipitation. A total of 21 meteorological stations including Ling county (37°20′N, 116°34′E), Huimin county (37°29′N, 117°32′E), Zhangqiu (36°41′N, 117°33′E), Kenli (37°36′N, 118°32′E), Changdao (37°56′N, 120°43′E), Longkou (37°37′N, 120°19′E), Fushan (37°30′N, 121°15′E), Chengshantou (37°24′N, 122°41′E), Shen county (36°14′N, 115°38′E), Jinan (36°36′N, 117°03′E), Yiyuan (36°11′N, 118°09′E), Pingdu (36°47′N, 120°00′E), Weifang (36°45′N, 119°11′E), Qingdao (36°04′N, 120°20′E), Haiyang (36°46′N, 121°11′E), Dingtao (35°06′N, 115°33′E), Yanzhou (35°34′N, 116°51′E), Fei county (35°15′N, 117°57′E), Ju county (35°35′N, 118°50′E), Rizhao (35°26′N, 119°32′E), and Xuzhou (34°28′N, 117°15′E) were used for our study. If a prefecture-level city has several meteorological stations, the average values of these meteorological stations were taken as the meteorological data of the prefecture-level city. Based on the principle of the closest distance and latitude, the missing meteorological data of Laiwu City, Zaozhuang City, and Taian City were filled by the averages of the weekly meteorological data of the neighboring stations Jinan and Yiyuan, Xuzhou, and Jinan and Yanzhou, respectively (22, 23). We obtained the air pollution data from a public weather website (http://www.tianqihoubao.com/). The air pollution data included particulate matter <2.5 μm in aerodynamic diameter (PM_2.5_), sulfur dioxide (SO_2_), and nitrogen dioxide (NO_2_). Weekly mean ambient temperatures, relative humidity, wind speed, PM_2.5_, SO_2_, and NO_2_ were obtained by calculating the average daily of these variables for a week. However, precipitation was the cumulative value of 7 days from Monday to Sunday.

### 2.3. Study design and statistical analysis

A two-stage analytical method (24) was used to analyze the effects of ambient temperature on ILI. In the first stage, the DLNM with quasi-Poisson distribution was applied to each prefecture-level city's data to obtain city-specific association estimates between ambient temperature and ILI. The DLNM can describe the complex non-linear and delayed relationship of temperature and ILI by a cross-basis function, which defines the traditional exposure–response relationship and the additional lag–response relationship, respectively (25). We used a natural cubic spline function with 7 degrees of freedom (df) per year to control the long-term trend and seasonality of ILI incidence (23, 26, 27). Several studies have found that relative humidity (28), wind speed (29), precipitation (30), PM_2.5_ (31), SO_2_ (32), and NO_2_ (33) were associated with ILI or influenza, and these factors were usually linked with the replication and sustained transmission of pathogens in the environment. Therefore, we used natural cubic splines with 3 df in DLNM for relative humidity, wind speed, precipitation, PM_2.5_, SO_2_, and NO_2_ to adjust for potential confounding based on previous literature (14, 23, 34, 35). To investigate the whole lag structure of the ambient temperature effect, the maximum lag was set at 3 weeks due to ILI incubation and duration (36). Autocorrelation function (ACF) and partial autocorrelation function (PACF) were used on the residuals (errors) from the model to check for the existence of autocorrelation. The model was described as follows:


$$\begin{array}{l} Log[E(Y_{t})]=\alpha +\beta temp_{t,l}+ns(time,7^{*}3)+ns(hum_{t},3)\\ \;+\;ns(wind_{t},3)+ns(rain_{t},3)+ns(PM_{2.5t},3)\\ \;+\;ns(NO_{2t},3)+ns(SO_{2t},3) \end{array}$$

where *Y*_*t*_ was the incidence of ILI in the week of study *t* (*t* = 1, 2, …, 169) in each prefecture city. α was the overall intercept. *temp*_*t,l*_ was the cross-matrices obtained by applying a basis function for ambient temperature and time through DLNM. We determined 4 df for both the response and lag dimensions of ambient temperature (34, 37–39). β was the coefficient of matrices. The notation *ns*(.) represented a natural cubic spline function. The median temperature of each prefecture-level city of the corresponding region was defined as the reference (22, 34) when calculating the overall cumulative relative risk (cRR). We used 5 and 95% of the weekly mean ambient temperature as extreme cold (*P*_5_) and extreme hot temperature (*P*_95_) (40).

In the second stage, we used a multivariate meta-analysis with a random-effects model to pool the estimates of city-specific exposure–response associations for overall cumulative, resulting in a best linear unbiased prediction (BLUP) between overall ambient temperature and ILI (41). The estimates can be assessed more accurately because the BLUP method is a sweet spot in the trade-off between city-specific associations and second-stage pooled estimates (42). We also combined the city-specific lag–response associations at extreme temperatures (*P*_5_ and *P*_95_) and obtained a BLUP between extreme temperature (*P*_5_ and *P*_95_) and ILI at the regional and provincial levels (43). The multivariate extension of Cochran's *Q-*test and *I*^2^ statistic was adopted to measure the residual heterogeneity (44, 45). In addition, stratification analyses by age were conducted to identify the potentially vulnerable groups.

To test the stability of the main findings, sensitivity analyses were conducted by changing df for time trend (6–8), df for weekly mean ambient temperature (3–6), df for the confounders (2–6), adjusting for the confounders changing different effect models (fixed-effect or random-effect) for cold and hot effects of ambient temperature and changing the average metrics and selection of stations which were used to replace the missing data in Taian, Laiwu, and Zaozhuang.

The analysis and modeling were all carried out with the R software (version 4.0.4, R Foundation for Statistical Computing) using the “dlnm” package to construct the DLNMs and the “mvmeta” package to perform the multivariate meta-analysis. A *P* ≤ 0.05 was considered to indicate statistical significance.

## 3. Results

### 3.1. Characteristics of ILI, meteorological data, and air pollution data

**Figure 1:**
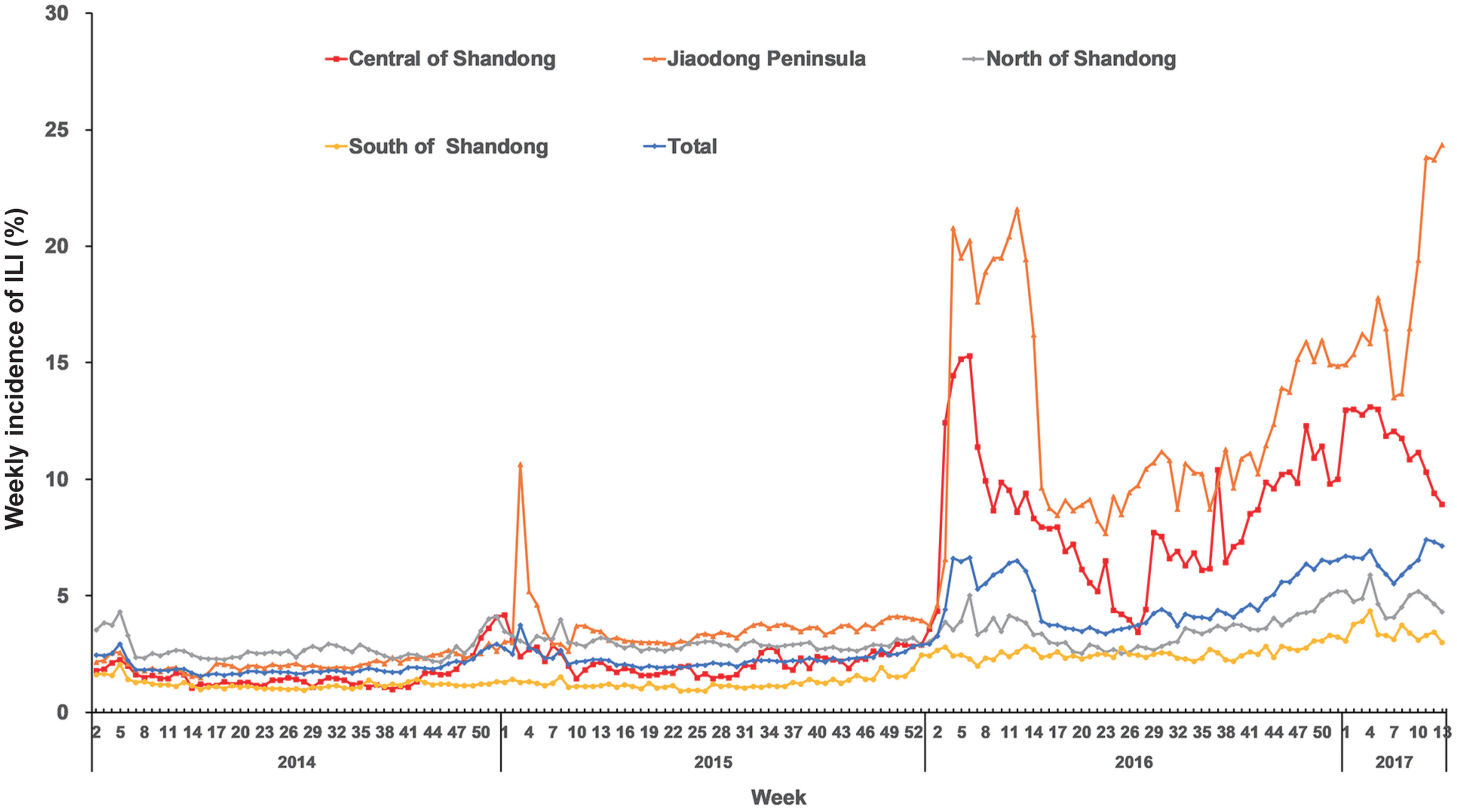
Region-specific and total weekly incidences of ILI in Shandong Province from 2014 to 2017.

The weekly incidences of ILI in 17 cities of Shandong Province during the study period are shown in Table 1. Sentinel hospitals for influenza surveillance reported 911,743 ILI cases (3.36%) during the study period and there were 27,117,379 hospital outpatients and emergency care visits. The highest median of weekly incidence of ILI was in Qingdao (6.24%), followed by Dezhou (4.62%), and the lowest median of weekly incidence of ILI was in Weihai (0.66%). In Shandong Province, there was a significant seasonality for ILI (Figure 1). The activity of ILI increased from autumn and peaked during winter and spring (from December to March of the following year) despite the large variations exhibited in the weekly peak intensity across the years, especially in the regions of Central Shandong and Jiaodong Peninsula. The highest weekly incidence of ILI occurred during the 11th week in 2017 (7.42%) in total.

**Table 1:** Weekly incidence of influenza-like illness (%) in each city of Shandong, China during 2014–2017.

**Cities**	**Mean ±SD**	**Minimum**	***P*_25_**	**Median**	***P*_75_**	**Maximum**
**Central Shandong**						
Jinan	4.61 ± 3.92	1.00	1.62	2.45	7.64	15.31
Taian	2.21 ± 0.71	1.03	1.71	2.12	2.51	5.76
Laiwu	2.98 ± 1.08	1.23	2.17	2.84	3.49	6.81
Zibo	2.63 ± 0.77	1.30	2.11	2.52	3.18	5.14
Weifang	2.94 ± 1.06	1.59	2.26	2.66	3.17	6.85
**Jiaodong Peninsula**						
Qingdao	12.42 ± 11.36	1.98	3.81	6.24	20.09	47.58
Yantai	2.61 ± 0.98	0.47	2.05	2.43	3.07	5.69
Weihai	0.87 ± 0.56	0.33	0.45	0.66	1.12	3.39
**North Shandong**						
Liaocheng	2.14 ± 1.24	0.79	1.28	1.78	2.35	6.26
Dezhou	4.92 ± 1.18	3.18	3.97	4.62	5.82	8.27
Binzhou	2.81 ± 0.74	1.53	2.27	2.66	3.22	5.34
Dongying	3.87 ± 0.77	2.43	3.37	3.76	4.21	7.60
**South Shandong**						
Heze	2.38 ± 0.66	1.15	1.94	2.34	2.81	4.15
Jining	1.20 ± 0.81	0.49	0.65	0.77	1.58	4.68
Zaozhuang	2.92 ± 1.07	1.31	2.03	2.56	3.89	6.60
Linyi	3.22 ± 1.59	1.33	1.82	2.33	5.02	6.15
Rizhao	1.37 ± 0.53	0.36	0.95	1.30	1.73	2.69

*P*_25_, the 25th percentile; *P*_75_, the 75th percentile; SD, standard deviation.

Supplementary Table 1 shows a summary of the descriptive statistics of the weekly meteorological data and air pollutant data of each city. The median of the weekly average ambient temperature during 2014–2017 in Shandong Province was 14.99°C (range: −8.30–31.69°C). The medians of the weekly average relative humidity, average wind speed, cumulative precipitation, PM_2.5_, NO_2_, and SO_2_ were 65.71% (range: 27.79–97.29%), 2.06 m/s (range: 0.77–8.90 m/s), 1.80 mm (range: 0–229.8 mm), 67.29 μg/m^3^ (range: 11.57–332.29 μg/m^3^), 37.79 μg/m^3^ (range: 7.29–91.14 μg/m^3^), and 39.43 μg/m^3^ (range: 7.29–91.14 μg/m^3^), respectively (data not shown).

### 3.2. The specific-city analysis of ambient temperature and ILI

**Figure 2:**
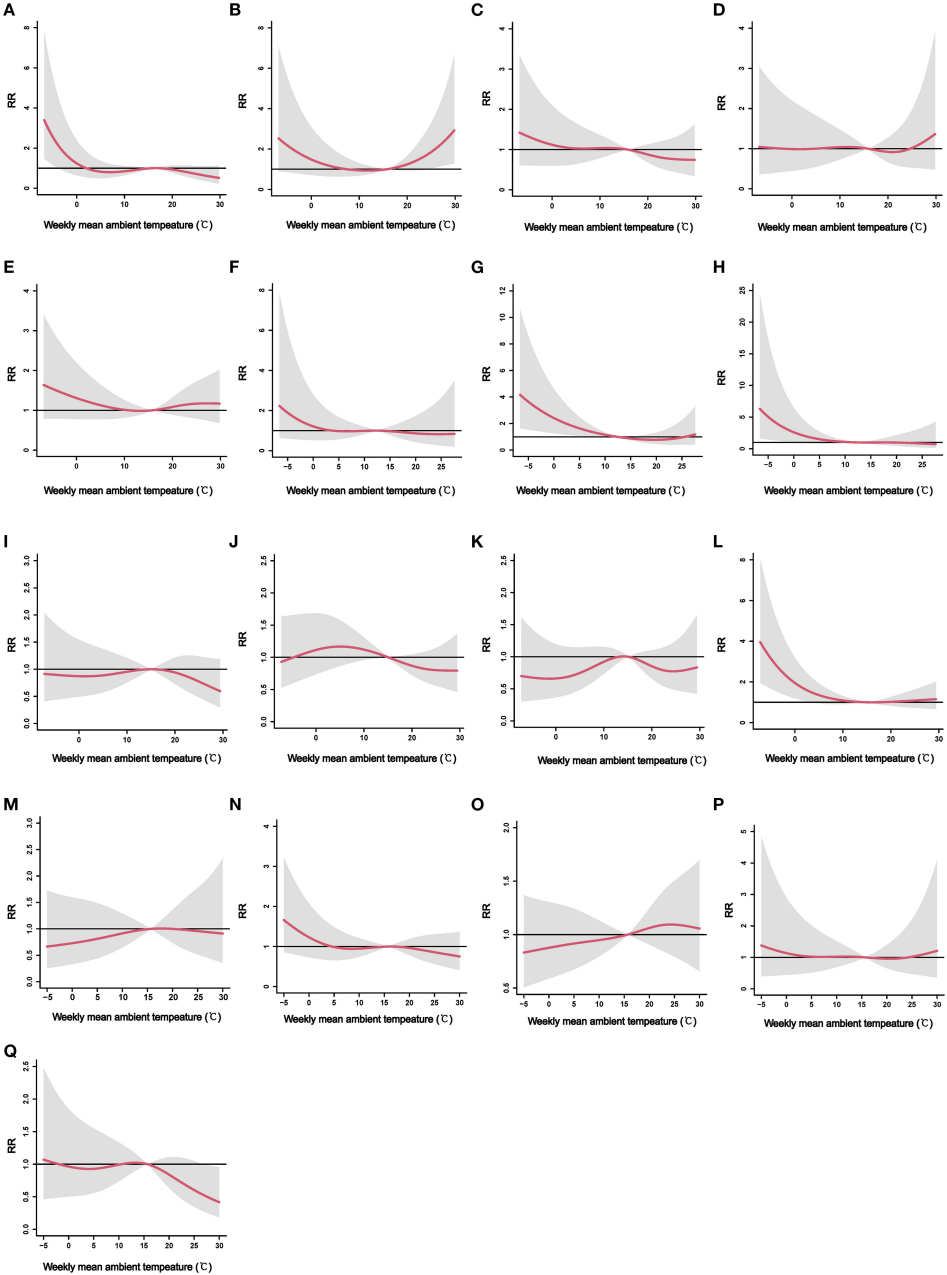
The city-specific exposure–response curves of the cumulative effects of weekly mean ambient temperature on ILI. ILI, influenza-like-illness; RR, relative risk. **(A)** Jinan, **(B)** Taian, **(C)** Laiwu, **(D)** Zibo, **(E)** Weifang, **(F)** Qingdao, **(G)** Yantai, **(H)** Weihai, **(I)** Liaocheng, **(J)** Dezhou, **(K)** Binzhou, **(L)** Dongying, **(M)** Jining, **(N)** Zaozhuang, **(O)** Linyi, **(P)** Rizhao, and **(Q)** Heze.

Figure 2 displays the city-specific exposure–response curves of the cumulative effects of ambient temperature on ILI from the DLNM models at the first stage. Although no significant cumulative effect of ambient temperature on ILI was found in Laiwu (Figure 2C), Zibo (Figure 2D), Weifang (Figure 2E), Qingdao (Figure 2F), Liaocheng (Figure 2I), Dezhou (Figure 2J), Binzhou (Figure 2K), Jining (Figure 2M), Zaozhuang (Figure 2N), Linyi (Figure 2O), and Rizhao (Figure 2P), the exposure–response curves showed a non-linear association between ambient temperature and ILI. However, the risk of ILI increased during cold ambient temperatures, especially below 0°C in Jinan (Figure 2A), Yantai (Figure 2G), Weihai (Figure 2H), and Dongying (Figure 2L). Although no cumulative effect of high ambient temperature on ILI was found in most cities, a high temperature was found to increase the risk of ILI in Taian (Figure 2B). In contrast, high ambient temperature (from 27°C) was found to decrease the risk of ILI in Heze (Figure 2Q). The three-dimensional (3D) plots show a visualized exposure–lag–response relationship between ambient temperature and the risk of ILI in each city (Supplementary Figure 2).

### 3.3. The region-specific analysis of ambient temperature and ILI

**Figure 3:**
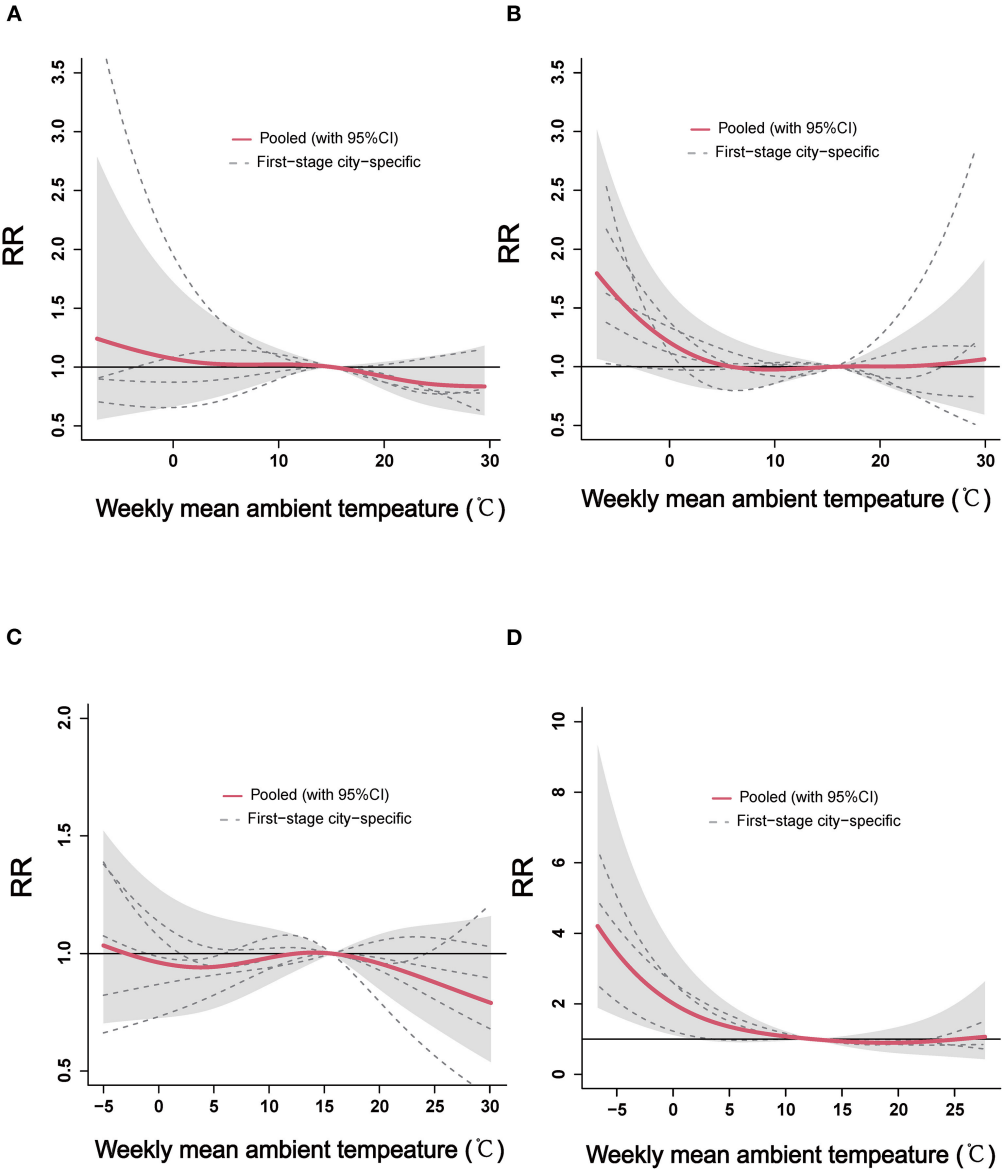
The region-specific pooled estimates of weekly mean ambient temperature on ILI in the four regions of Shandong Province. ILI, influenza-like-illness; RR, relative risk. **(A)** North Shandong, **(B)** Central Shandong, **(C)** South Shandong, and **(D)** Jiaodong Peninsula.

The region-specific exposure–response curves of cumulative effects of ambient temperature on ILI for the four regions are demonstrated in Figure 3. The region-specific analyses suggested a non-linear relationship between weekly mean ambient temperature and ILI: an approximate “L”-shaped relationship for North Shandong (Figure 3A), Central Shandong (Figure 3B), and Jiaodong Peninsula (Figure 3D), while an approximate “S”-shaped relationship for South Shandong (Figure 3C). The risk of ILI was significantly increased in association with cold ambient temperatures in Central Shandong and Jiaodong Peninsula regions (Figures 3B, D). When the weekly mean ambient temperature was below −4°C, the pooled effect became statistically significant in Central Shandong (RR = 1.50, 95% CI: 1.00–2.25). While in Jiaodong Peninsula, the effect of exposure to an ambient temperature below 1.9°C became significant (RR = 1.69, 95% CI: 1.00–2.86). However, the risk of ILI was not significantly associated with the weekly mean ambient temperature in the pooled analysis in North and South Shandong (Figures 3A, C).

The cold and hot effects of ambient temperature on ILI are displayed in Table 2. In Jiaodong Peninsula, statistical significances of the cold effects were found lagging at weeks 1 (RR = 1.28, 95% CI: 1.01–1.62) and 3 (RR = 1.30, 95% CI: 1.06–1.60). In Central and North Shandong, the cold effects of ambient temperature significantly lagged at weeks 3 (RR = 1.15, 95% CI: 1.00–1.32) and 0 (RR = 0.86, 95% CI: 0.75–0.98), respectively. Instead, there was no association between lagging at different weeks and the cold effects of ambient temperature in South Shandong. For the hot effect of ambient temperature on ILI, the relationship between extremely high temperature and ILI was not significant in all four regions.

**Table 2:** The cold and hot effects with reference to temperature of 50th on influenza-like illness in Shandong, China.

**Lag**	**Central Shandong**		**Jiaodong Peninsula**		**North Shandong**		**South Shandong**	
	**RR**	**95% CI**	**RR**	**95% CI**	**RR**	**95% CI**	**RR**	**95% CI**
**Cold effect (** *P*_5_ **)**								
Lag 0	1.01	0.88–1.17	1.01	0.76–1.34	0.86	0.75–0.98^*^	1.05	0.88–1.24
Lag 1	1.04	0.89–1.22	1.28	1.01–1.62^*^	1.02	0.82–1.26	0.96	0.86–1.08
Lag 2	1.07	0.89–1.29	1.25	0.90–1.72	1.12	0.94–1.33	0.97	0.86–1.10
Lag 3	1.15	1.00–1.32^*^	1.30	1.06–1.60^*^	1.14	0.93–1.39	0.98	0.88–1.08
**Hot effect (** *P*_95_ **)**								
Lag 0	0.97	0.77–1.20	0.83	0.58–1.18	0.91	0.78–1.07	1.05	0.93–1.20
Lag 1	1.03	0.91–1.17	1.01	0.76–1.36	0.97	0.83–1.12	0.89	0.79–1.01
Lag 2	1.05	0.92–1.19	1.08	0.81–1.45	0.96	0.84–1.10	0.93	0.81–1.08
Lag 3	1.01	0.84–1.21	1.00	0.75–1.35	1.00	0.87–1.16	1.01	0.89–1.14

^*^P < 0.05. CI, confidence interval; RR, relative risk.

In addition, the heterogeneity of the random-effect meta-analysis of each region in the second stage is shown in Supplementary Table 2. The heterogeneity among the regions of Central Shandong (*I*^2^ = 64.39%) and North Shandong (*I*^2^ = 74.87%) was significant. There was no significant heterogeneity among the regions of Jiaodong Peninsula (*I*^2^ = 13.33%) and South Shandong (*I*^2^ = 17.73%).

### 3.4. The overall pooled analysis of ambient temperature and ILI

**Figure 4:**
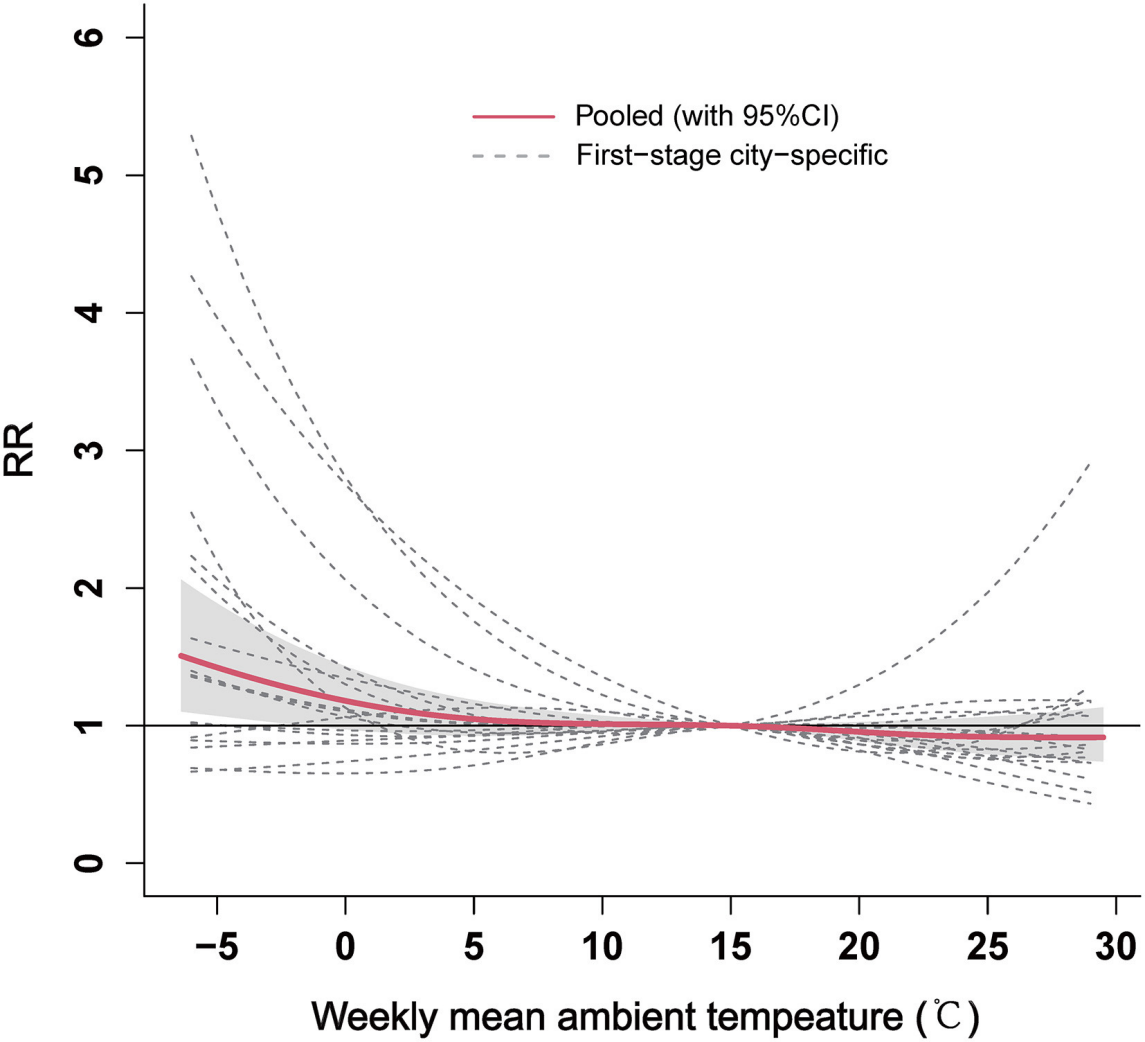
The overall pooled estimates between weekly mean ambient temperature and ILI for the 17 cities in Shandong Province. ILI, influenza-like-illness; RR, relative risk.

Figure 4 shows the overall pooled association between weekly mean ambient temperature and ILI for the 17 cities in Shandong Province. The relationship between ambient temperature and ILI presented an approximately slow “L” shape at the provincial level. The significant heterogeneity between the city-specific relationships was observed with an *I*^2^ of 57.59% (*Q*-test: *P* < 0.01, Supplementary Table 2). A gradual decrease in the risk of ILI with increasing ambient temperature was observed, and the effect was statistically significant when the temperatures were <-1.5°C (RR = 1.24, 95% CI: 1.00–1.54). In addition, the stratification analyses suggested that the effects of low temperature on ILI presented a statistical significance in the 5–14, 15–24, and 25–59 year age groups (Supplementary Figures 3B–D), while no significant cumulative effect of low temperature on ILI was found in the 0–4 and ≥60 year age groups (Supplementary Figures 3A, E).

**Figure 5:**
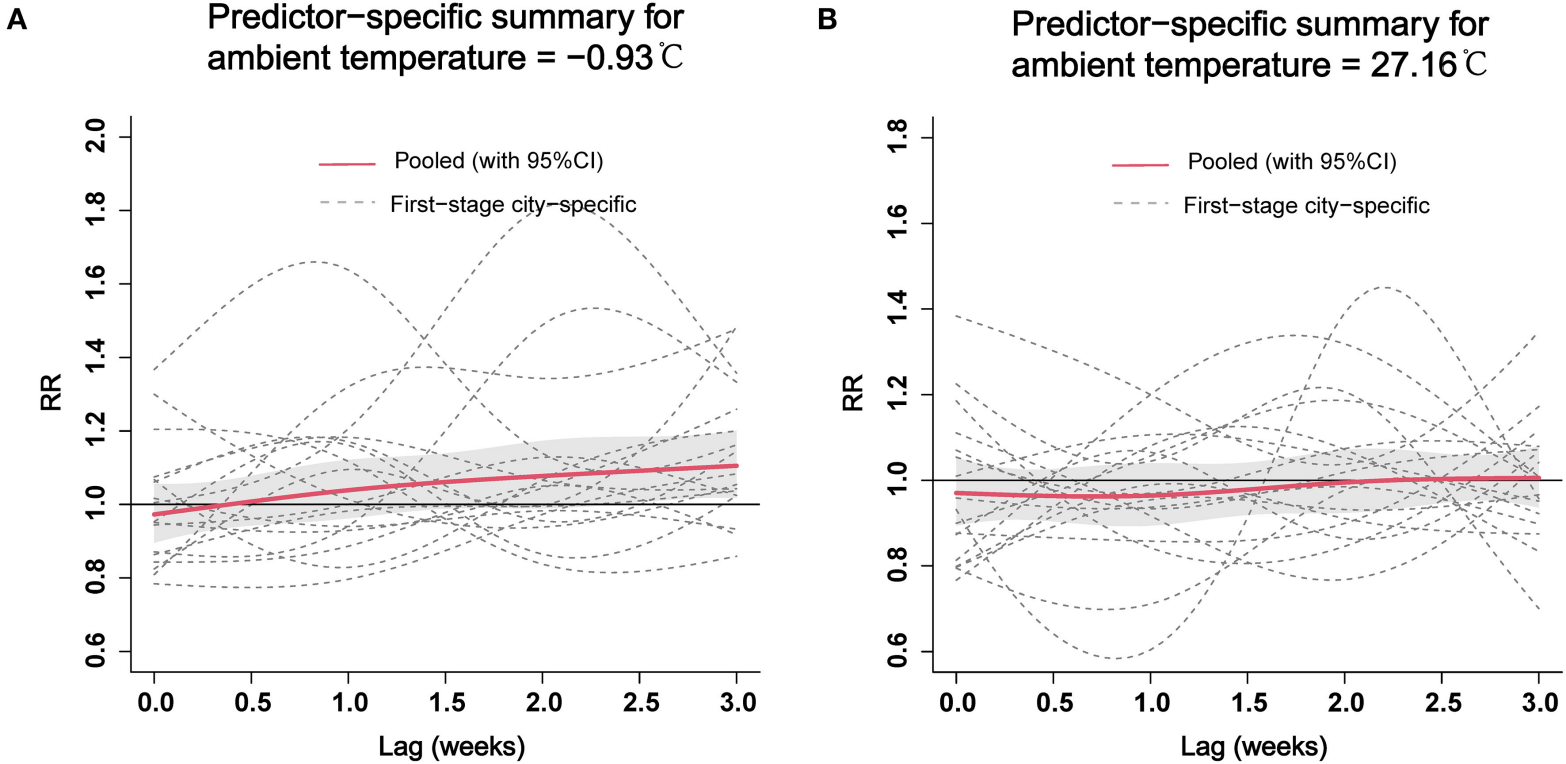
The pooled estimates of specific lag–response curves of cold effect **(A)** and hot effect **(B)** on ILI at the provincial level. ILI, influenza-like-illness; RR, relative risk.

We also analyzed the pooled of specific lag-response curves of cold effect (−0.93 °C) and hot effect (27.16 °C) at provincial level, which are represented in Figure 5. The risk of ILI seemed an increasing trend for cold effect and showed statistical significance from 2.5 to 3 weeks. However, the hot effect of ambient temperature was not be statistically significant at different lag weeks.

### 3.5. Sensitivity analysis

The robustness of our pooled effects was tested using sensitivity analyses. The results of the association between ambient temperature and ILI were similar when changing the df for time trend and ambient temperature (Supplementary Figure 4), changing the df for confounders and adjusting for the confounders (Supplementary Figure 5), and changing different effect models (Supplementary Figure 6). The cumulative effect estimates from the sensitivity analyses of Taian, Laiwu, and Zaozhuang showed stable results when changing the average metrics and the selection of stations which were used to replace the missing data (Supplementary Figure 7). Supplementary Figures 8, 9 show there were no obvious autocorrelations from each city-specific DLNM model. We observed that residuals for the DLNM models followed a normal distribution (Supplementary Figure 10).

## 4. Discussion

In this study, first, we quantitatively elucidate the association between ambient temperature and ILI at the city, regional, and provincial levels in Shandong Province, China. Based on the results of the study after controlling for potential confounders, it was found that lower ambient temperature was statistically significantly associated with a higher risk of ILI at the provincial level and identified the effect of cold on the risk of ILI in the study area. Climate change is currently considered to be one of the major threats to public health (1). In the future, extreme weather events may occur more frequently caused by climate change (46). With the increasing frequency of extreme temperature events, ILI outbreaks may happen during the occurrence of extremely low temperatures in the future, which can impose a severe burden on the healthcare system. In addition, management and prevention interventions should be made for ILI when low temperature occurs. Officials would benefit from knowing how ambient temperature affects ILI to optimize the allocation of healthcare resources in consideration of extreme weather as a factor.

Our results indicate that ILI activity presents a seasonal trend with a peak during winter and spring, which suits the view that the epidemic season fastens in winter and spring in China's northern areas (47). The seasonality of ILI may be associated with environmental conditions (e.g., ambient temperature) that can affect the immune system and facilitate the transmission of the pathogen. Although the weekly incidence of ILI changed from city to city, high incidences of ILI were observed in the Jiaodong Peninsula and Central Shandong since 2016, especially in Qingdao and Jinan. The reason for the high incidence of ILI in Qingdao and Jinan cannot be identified in our study. It may be because Qingdao and Jinan have become active surveillance sites for ILI under the CISIS since 2016.

This study has identified that low ambient temperature is significantly associated with ILI at the provincial level. The low temperature, in agreement with previous studies, is the risk factor for ILI (3, 6, 21). Different hypotheses have been suggested to explain the phenomenon that low temperature leads to more ILI cases, including influenza. A study by Eccles showed that cold air inhalation chills the nasal epithelium and weakens the immune system against respiratory influenza infection (48). Vitamin D is recognized as the sunshine vitamin which can prevent respiratory infection by elevating antimicrobial peptide levels (AMPs). This effect may be considered to alter the normal immunity horizontal through vitamin D, which is directly associated with daylight (11, 49, 50). The phenomenon conforms to the result that people have observed: the common cold and influenza are most frequently observed in winter and spring, while vitamin D levels are lowest during these two seasons. Furthermore, some studies also showed that both cold and dry air has proven to be very beneficial for viral survival (51, 52). Cold may extend viral particle survival and people tend to stay indoors with crowding, which resulted in increased exposure. Crowding has been postulated as a risk factor in diseases that are caused by bacteria and viruses, including influenza (52). Meanwhile, the pathogens causing ILI have more than one species of viruses, which can increase the possibility of infection of the susceptible population through person-to-person contact and/or airborne transmission.

The results suggest that low ambient temperatures significantly increase the risk of ILI in the Jiaodong Peninsula and Central Shandong at the regional level, while no such effect was observed in other regions. The reason may be that, in addition to the effect of low temperatures, ILI could be influenced by other factors such as low relative humidity (53, 54) and high wind speed (29). The lower relative humidity in Central Shandong and higher wind speed in the Jiaodong Peninsula align with this view. Similar results are also observed at the city level. The data from the laboratory guinea pig as a model host indicate that low relative humidity and low temperature are favorable for virus survival and aerosol transmission (12, 51). Another study showed that the risk of ILI was increased by low air temperature, low absolute humidity, and high wind speed (29). Wind speed is also a contributing factor for virus dispersal (55). In addition, there was a certain lag effect of cold on ILI in Central Shandong and the Jiaodong Peninsula. We considered that indoor crowding during cold weather, seasonal fluctuations in host immune responses, and environmental factors were responsible for the lag effect of cold.

However, our study indicates that high temperatures had no significant effect on the risk of ILI at the regional and provincial levels, including the hot effect. A study conducted in Guilin of China showed that there was no significant association between thermal effect and influenza (15), which is consistent with our study. Some studies showed that elevated temperature had a negative correlation with risk the influenza (17, 36, 56). This is probably because higher temperatures (20–30°C) may have prevented the aerosol transmission, contact, and short-distance spread of influenza (57). However, an Australian study showed that a higher risk of infection was caused by high temperatures (>28°C) (58). The direct or indirect relationship could be confirmed between the increased use of air conditioning in summer and the peak of influenza in summer (59). People may stay in an air conditioning environment under high temperatures, both cooler and drier conditions, leading to an influenza epidemic. However, controversy in this view, using air conditioning would lower the indoor absolute humidity through the condensation, which may trap virus-bearing aerosols within the unit itself (52).

In addition, there was significant heterogeneity among the city-specific relationships when obtaining the overall cumulative exposure–response relationship at the regional (Central Shandong and North Shandong) and provincial levels. The heterogeneity may come from diverse ecological and geographical characteristics. The differences in geographical characteristics could have contributed to city-specific temperature–ILI relationships. For example, the wind speed is higher in winter in coastal cities than those in inland cities. Due to the high wind speed, airborne aerosols travel further, which may contribute to the transmission of ILI/influenza pathogens (37).

There are some limitations to this study, which should be acknowledged. First, the relationship between ambient temperature and ILI was analyzed at a city-specific level, which may result in an ecological fallacy. In particular, for meteorological data, we used ambient temperature from the observation stations to reflect individual exposure. Due to the missing meteorological data, the meteorological data of three cities from the neighboring stations or the average of the neighboring stations were used for the calculations. These might be some of the main causes of the ecological fallacy because the temperature was not an individual exposure. Second, our data on the ILI are in weeks, which only estimates the weekly lag effects, when investigating the exposure–response relationship, which is not as accurate as the estimation of the daily lag effect. Third, some confounding factors like different pathogens of ILI, health-seeking behaviors, immune levels, vaccination, and availability of health services have not been considered to control. Fourth, we failed to analyze the effect of ambient temperature on ILI from 2017 to 2022, which might be slightly different from 2014 to 2017, because the Shandong Center for Disease Control and Prevention only shared with us the data from 2014 to 2017. Finally, the underreporting bias is inevitable, because patients often avoid medical help immediately, which leads to an underestimation of the reported cases during the cold winter days.

## 5. Conclusion

Our study presents the first comprehensive assessment of the relationship between ambient temperature and ILI in China using a two-stage analytical method at the provincial level. Our findings support that low temperatures can increase the risk of ILI. In addition, the cold effect of ambient temperature on ILI is consistently stronger than that of the hot effect. Meanwhile, our findings provide useful information for instituting early warning systems for ILI and developing strategies to respond to climate change.

## Data availability statement

The raw data supporting the conclusions of this article will be made available by the authors, without undue reservation.

## Author contributions

GD, XW, and WX designed the study and supervised the study. TL, LS, SS, SZ, JW, and ZL collated the data. JY, FT, and DC analyzed the data. JY and GD drafted the manuscript. All authors had input on the manuscript draft, interpreted the results, revised the report, and approved the final version.
