# Supplementary material for: Effects of ambient temperature on influenza-like illness: A multicity analysis in Shandong Province, China, 2014–2017
Source: Front Public Health. 2023 Jan 9;10:1095436. doi: 10.3389/fpubh.2022.1095436 (PMC9868675; doi:10.3389/fpubh.2022.1095436)
Supplement: Supplementary file 1 [file Data_Sheet_1.docx]

**Supplementary Material**

**Effects of ambient temperature on influenza-like illness: A multicity analysis in Shandong Province, China, 2014-2017**

Jia Yin, Ti Liu, Fang Tang, Dongzhen Chen, Lin Sun, Shaoxia Song, Shengyang Zhang, Julong Wu, Zhong Li, Weijia Xing, Xianjun Wang, Guoyong Ding

**Supplemental Tables and Figures**

| **Contents** | **Pages** |
| --- | --- |
| **Supplementary Table 1.** Descriptive statistics for weekly meteorological data and air pollutant data during the study period | 3 |
| **Supplementary Table 2.** Tests of heterogeneity for second-stage in multivariate meta-analyses | 8 |
| **Supplementary Figure 1.** Location of the study area in China and geographic regions of Shandong Province | 9 |
| **Supplementary Figure 2.** Three-dimensional (3D) exposure-lag-response plot for weekly mean ambient temperature and ILI in 17 cities | 10 |
| **Supplementary Figure 3.** The overall pooled estimate between weekly mean ambient temperature and ILI in different age groups | 11 |
| **Supplementary Figure 4.** Pooled estimates of weekly ambient temperature on ILI when changing the df for time (A) and ambient temperature (B) | 12 |
| **Supplementary Figure 5.** Pooled estimates of weekly ambient temperature on ILI when changing the df for confounders (A) and adjusting for confounders (B) | 13 |
| **Supplementary Figure 6.** Pooled estimates of weekly ambient temperature on ILI when changing the different effect models | 14 |
| **Supplementary Figure 7.** The city-specific cumulative effects of weekly mean ambient temperature on ILI when changing average metrics and selection of stations to replace missing data | 15 |
| **Supplementary Figure 8.** Autocorrelation function (ACF) plots of residuals in 17 cities derived from the city-specific DLNM models | 16 |
| **Supplementary Figure 9.** Partial autocorrelation function (PACF) of residuals in 17 cities derived from the city-specific DLNM models | 17 |
| **Supplementary Figure 10.** Histogram of residuals in 17 cities derived from the city-specific DLNM models | 18 |

**Supplementary Table 1.** Descriptive statistics for weekly meteorological data and air pollutant data during the study period.

| Variables | Mean±SD | Minimum | *P*_25_ | Median | *P*_75_ | Maximum |
| --- | --- | --- | --- | --- | --- | --- |
| **Central Shandong** |  |  |  |  |  |  |
| Jinan |  |  |  |  |  |  |
| Ambient temperature (℃) | 14.38±10.00 | -6.06 | 4.39 | 16.58 | 23.76 | 30.61 |
| Relative humidity (%) | 56.50±14.30 | 27.79 | 44.29 | 56.14 | 68.29 | 87.79 |
| Wind speed (m/s) | 2.27±0.61 | 1.21 | 1.84 | 2.14 | 2.60 | 4.26 |
| Cumulative precipitation (mm) | 11.92±20.82 | 0 | 0 | 2.50 | 11.95 | 113.00 |
| PM_2.5_ (μg/m^3^) | 84.84±35.55 | 36.29 | 61.29 | 77.00 | 100.00 | 254.14 |
| NO_2_ (μg/m^3^) | 50.77±13.86 | 26.57 | 38.71 | 49.57 | 59.79 | 87.71 |
| SO_2_ (μg/m^3^) | 48.90±27.31 | 14.71 | 28.29 | 41.86 | 60.45 | 141.71 |
| Taian |  |  |  |  |  |  |
| Ambient temperature (℃) | 14.21±9.86 | -6.27 | 4.73 | 16.23 | 23.59 | 30.44 |
| Relative humidity (%) | 63.17±13.08 | 36.36 | 54.04 | 62.00 | 73.54 | 90.29 |
| Wind speed (m/s) | 1.91±0.45 | 1.04 | 1.56 | 1.82 | 2.23 | 3.33 |
| Cumulative precipitation (mm) | 6.79±11.42 | 0 | 0 | 1.79 | 8.21 | 58.07 |
| PM_2.5_ (μg/m^3^) | 70.41±31.58 | 27.00 | 48.07 | 63.29 | 84.36 | 194.29 |
| NO_2_ (μg/m^3^) | 41.37±12.51 | 18.14 | 32.86 | 39.86 | 47.86 | 81.00 |
| SO_2_ (μg/m^3^) | 41.30±20.26 | 11.43 | 27.79 | 35.29 | 52.50 | 126.71 |
| Laiwu |  |  |  |  |  |  |
| Ambient temperature (℃) | 13.78±9.88 | -7.09 | 4.03 | 15.92 | 23.04 | 29.74 |
| Relative humidity (%) | 58.70±14.02 | 30.43 | 48.57 | 57.93 | 69.64 | 89.21 |
| Wind speed (m/s) | 2.02±0.46 | 0.96 | 1.67 | 1.96 | 2.32 | 3.46 |
| Cumulative precipitation (mm) | 6.26±10.99 | 0 | 0 | 1.85 | 7.63 | 69.45 |
| PM_2.5_ (μg/m^3^) | 85.73±33.27 | 30.71 | 62.79 | 79.43 | 104.93 | 200.71 |
| NO_2_ (μg/m^3^) | 48.69±15.33 | 14.29 | 38.17 | 46.57 | 58.21 | 89.29 |
| SO_2_ (μg/m^3^) | 63.02±35.02 | 6.71 | 37.79 | 55.71 | 82.93 | 189.86 |
| Zibo |  |  |  |  |  |  |
| Ambient temperature (℃) | 12.96±9.93 | -7.81 | 3.24 | 14.99 | 22.19 | 28.86 |
| Relative humidity (%) | 61.45±13.62 | 30.29 | 51.71 | 61.86 | 72.93 | 89.14 |
| Wind speed (m/s) | 1.70±0.43 | 0.89 | 1.40 | 1.64 | 1.98 | 3.01 |
| Cumulative precipitation (mm) | 10.59±19.05 | 0 | 0 | 2.90 | 13.60 | 112.80 |
| PM_2.5_ (μg/m^3^) | 84.67±32.89 | 38.14 | 62.07 | 78.00 | 97.25 | 229.57 |
| NO_2_ (μg/m^3^) | 58.65±12.17 | 27.43 | 50.43 | 57.79 | 66.75 | 88.43 |
| SO_2_ (μg/m^3^) | 82.07±35.79 | 14.14 | 55.21 | 74.21 | 104.79 | 185.71 |
| Weifang |  |  |  |  |  |  |
| Ambient temperature (℃) | 13.56±10.20 | -7.56 | 3.11 | 15.43 | 22.89 | 30.24 |
| Relative humidity (%) | 61.96±11.71 | 36.00 | 54.43 | 62.14 | 71.00 | 86.43 |
| Wind speed (m/s) | 1.96±0.47 | 0.83 | 1.61 | 1.96 | 2.27 | 3.59 |
| Cumulative precipitation (mm) | 9.43±15.95 | 0 | 0 | 1.60 | 10.95 | 74.4 |
| PM_2.5_ (μg/m^3^) | 71.92±31.80 | 26.57 | 51.86 | 64.00 | 86.79 | 233.14 |
| NO_2_ (μg/m^3^) | 36.48±11.84 | 15.71 | 28.00 | 34.86 | 44.00 | 67.86 |
| SO_2_ (μg/m^3^) | 44.52±27.18 | 10.43 | 24.86 | 35.00 | 58.79 | 142.00 |
| **Jiaodong Peninsula** |  |  |  |  |  |  |
| Qingdao |  |  |  |  |  |  |
| Ambient temperature (℃) | 12.81±9.56 | -7.07 | 3.41 | 12.71 | 21.87 | 28.59 |
| Relative humidity (%) | 67.55±9.96 | 36.48 | 59.60 | 67.71 | 75.17 | 85.76 |
| Wind speed (m/s) | 2.51±0.50 | 1.40 | 2.15 | 2.46 | 2.78 | 3.90 |
| Cumulative precipitation (mm) | 9.71±18.21 | 0 | 0.13 | 2.03 | 12.13 | 173.63 |
| PM_2.5_ (μg/m^3^) | 49.84±24.83 | 14.00 | 33.07 | 45.43 | 59.64 | 184.14 |
| NO_2_ (μg/m^3^) | 34.99±11.95 | 10.29 | 25.86 | 33.57 | 41.43 | 70.14 |
| SO_2_ (μg/m^3^) | 26.12±14.06 | 5.43 | 17.36 | 21.57 | 30.79 | 85.56 |
| Yantai |  |  |  |  |  |  |
| Ambient temperature (℃) | 12.77±9.77 | -7.15 | 2.94 | 13.14 | 22.13 | 28.45 |
| Relative humidity (%) | 85.95±9.58 | 42.29 | 56.60 | 64.19 | 71.64 | 85.95 |
| Wind speed (m/s) | 3.41±0.74 | 1.86 | 2.83 | 3.32 | 3.92 | 6.25 |
| Cumulative precipitation (mm) | 9.14±19.44 | 0 | 0.05 | 2.10 | 8.33 | 147.07 |
| PM_2.5_ (μg/m^3^) | 44.90±20.65 | 15.86 | 30.21 | 42.00 | 54.07 | 143.57 |
| NO_2_ (μg/m^3^) | 35.67±10.11 | 16.14 | 28.36 | 34.86 | 42.07 | 66.14 |
| SO_2_ (μg/m^3^) | 23.32±11.60 | 7.14 | 14.14 | 20.14 | 30.57 | 68.14 |
| Weihai |  |  |  |  |  |  |
| Ambient temperature (℃) | 11.80±8.71 | -6.10 | 3.01 | 11.30 | 20.39 | 26.36 |
| Relative humidity (%) | 73.74±10.86 | 53.71 | 64.71 | 72.57 | 81.71 | 97.29 |
| Wind speed (m/s) | 5.18±1.29 | 2.07 | 4.33 | 5.23 | 5.99 | 8.90 |
| Cumulative precipitation (mm) | 9.75±26.46 | 0 | 0 | 1.00 | 9.40 | 229.8 |
| PM_2.5_ (μg/m^3^) | 37.15±16.17 | 11.57 | 26.43 | 34.86 | 47.29 | 128.57 |
| NO_2_ (μg/m^3^) | 22.55±8.22 | 7.29 | 16.07 | 21.43 | 28.21 | 55.00 |
| SO_2_ (μg/m^3^) | 16.51±8.14 | 4.57 | 11.36 | 14.29 | 19.85 | 56.14 |
| **North Shandong** |  |  |  |  |  |  |
| Liaocheng |  |  |  |  |  |  |
| Ambient temperature (℃) | 13.84±9.91 | -5.37 | 4.20 | 14.43 | 23.34 | 29.71 |
| Relative humidity (%) | 65.94±12.93 | 36.14 | 55.43 | 65.71 | 76.50 | 96.71 |
| Wind speed (m/s) | 2.01±0.53 | 0.90 | 1.66 | 1.97 | 2.34 | 3.53 |
| Cumulative precipitation (mm) | 10.01±19.37 | 0 | 0 | 1.30 | 11.50 | 142.60 |
| PM_2.5_ (μg/m^3^) | 94.14±38.98 | 42.29 | 67.29 | 84.57 | 112.29 | 280.29 |
| NO_2_ (μg/m^3^) | 43.52±14.75 | 21.14 | 30.86 | 40.29 | 54.71 | 84.14 |
| SO_2_ (μg/m^3^) | 40.22±21.48 | 5.71 | 25.50 | 35.43 | 53.14 | 114.00 |
| Dezhou |  |  |  |  |  |  |
| Ambient temperature (℃) | 13.02±10.36 | -7.80 | 2.76 | 14.56 | 22.86 | 29.07 |
| Relative humidity (%) | 67.00±12.72 | 36.86 | 57.29 | 66.71 | 77.21 | 93.86 |
| Wind speed (m/s) | 2.25±0.64 | 0.94 | 1.70 | 2.20 | 2.64 | 4.41 |
| Cumulative precipitation (mm) | 8.59±18.70 | 0 | 0 | 0.60 | 8.10 | 157.00 |
| PM_2.5_ (μg/m^3^) | 95.58±45.20 | 33.14 | 65.66 | 82.57 | 115.93 | 332.29 |
| NO_2_ (μg/m^3^) | 42.34±15.27 | 17.86 | 31.00 | 38.86 | 53.71 | 85.57 |
| SO_2_ (μg/m^3^) | 43.10±22.36 | 9.00 | 28.21 | 36.40 | 52.86 | 112.29 |
| Binzhou |  |  |  |  |  |  |
| Ambient temperature (℃) | 13.25±10.33 | -8.30 | 2.63 | 15.00 | 22.82 | 29.31 |
| Relative humidity (%) | 63.15±12.55 | 34.43 | 53.71 | 62.71 | 72.57 | 89.86 |
| Wind speed (m/s) | 2.09±0.53 | 1.06 | 1.70 | 2.03 | 2.46 | 3.67 |
| Cumulative precipitation (mm) | 11.73±29.65 | 0 | 0 | 1.20 | 9.90 | 224.80 |
| PM_2.5_ (μg/m^3^) | 79.84±30.99 | 30.86 | 57.29 | 77.00 | 92.29 | 238.14 |
| NO_2_ (μg/m^3^) | 41.12±14.58 | 15.00 | 30.00 | 39.14 | 51.79 | 83.86 |
| SO_2_ (μg/m^3^) | 52.58±26.16 | 12.00 | 35.57 | 47.00 | 64.36 | 154.57 |
| Dongying |  |  |  |  |  |  |
| Ambient temperature (℃) | 13.72±10.42 | -7.49 | 3.21 | 15.24 | 23.58 | 30.23 |
| Relative humidity (%) | 61.10±11.51 | 37.86 | 53.57 | 61.29 | 69.14 | 88.57 |
| Wind speed (m/s) | 2.12±0.46 | 1.16 | 1.73 | 2.10 | 2.45 | 3.69 |
| Cumulative precipitation (mm) | 11.21±25.14 | 0 | 0 | 1.00 | 9.90 | 168.70 |
| PM_2.5_ (μg/m^3^) | 73.57±32.61 | 22.57 | 52.14 | 68.00 | 86.64 | 262.14 |
| NO_2_ (μg/m^3^) | 41.43±14.76 | 15.29 | 29.71 | 40.83 | 51.21 | 85.86 |
| SO_2_ (μg/m^3^) | 56.55±21.89 | 16.57 | 42.00 | 51.86 | 69.43 | 136.14 |
| **South Shandong** |  |  |  |  |  |  |
| Heze |  |  |  |  |  |  |
| Ambient temperature (℃) | 14.02±9.55 | -4.31 | 4.93 | 15.79 | 23.49 | 30.14 |
| Relative humidity (%) | 69.37±12.33 | 41.57 | 59.21 | 69.71 | 80.14 | 93.14 |
| Wind speed (m/s) | 2.28±0.54 | 1.21 | 1.88 | 2.26 | 2.64 | 3.91 |
| Cumulative precipitation (mm) | 12.02±24.44 | 0 | 0 | 2.30 | 14.60 | 156.70 |
| PM_2.5_ (μg/m^3^) | 91.24±42.22 | 29.57 | 60.71 | 79.57 | 111.79 | 267.29 |
| NO_2_ (μg/m^3^) | 39.14±13.67 | 11.86 | 28.00 | 37.29 | 48.07 | 79.43 |
| SO_2_ (μg/m^3^) | 41.20±18.71 | 13.00 | 28.00 | 38.71 | 47.64 | 118.14 |
| Jining |  |  |  |  |  |  |
| Ambient temperature (℃) | 13.82±9.90 | -6.19 | 4.21 | 15.86 | 23.46 | 30.27 |
| Relative humidity (%) | 70.38±11.87 | 40.57 | 60.36 | 71.86 | 79.79 | 93.86 |
| Wind speed (m/s) | 1.47±0.42 | 0.77 | 1.17 | 1.38 | 1.77 | 2.50 |
| Cumulative precipitation (mm) | 11.65±20.33 | 0 | 0 | 2.80 | 14.00 | 111.30 |
| PM_2.5_ (μg/m^3^) | 78.45±34.98 | 31.29 | 55.57 | 69.71 | 93.79 | 224.29 |
| NO_2_ (μg/m^3^) | 43.77±13.75 | 12.57 | 32.57 | 42.43 | 53.21 | 79.71 |
| SO_2_ (μg/m^3^) | 54.51±24.72 | 15.00 | 37.43 | 50.57 | 66.14 | 143.57 |
| Zaozhuang |  |  |  |  |  |  |
| Ambient temperature (℃) | 14.95±9.40 | -3.14 | 6.08 | 16.73 | 23.55 | 31.69 |
| Relative humidity (%) | 67.50±11.32 | 38.43 | 58.86 | 68.71 | 76.36 | 90.29 |
| Wind speed (m/s) | 1.70±0.40 | 0.81 | 1.40 | 1.70 | 1.99 | 3.00 |
| Cumulative precipitation (mm) | 2.20±3.24 | 0 | 0 | 0.68 | 3.01 | 17.83 |
| PM_2.5_ (μg/m^3^) | 83.34±35.26 | 28.57 | 56.93 | 76.71 | 102.50 | 203.71 |
| NO_2_ (μg/m^3^) | 33.99±11.63 | 12.14 | 24.86 | 33.00 | 41.36 | 80.29 |
| SO_2_ (μg/m^3^) | 50.42±21.22 | 15.00 | 34.14 | 48.86 | 61.43 | 121.71 |
| Linyi |  |  |  |  |  |  |
| Ambient temperature (℃) | 14.13±9.65 | -5.51 | 4.82 | 15.93 | 23.01 | 30.00 |
| Relative humidity (%) | 64.37±12.26 | 30.00 | 55.71 | 65.29 | 74.36 | 86.43 |
| Wind speed (m/s) | 1.56±0.37 | 0.79 | 1.29 | 1.56 | 1.79 | 2.71 |
| Cumulative precipitation (mm) | 12.29±17.82 | 0 | 0 | 3.40 | 18.80 | 82.00 |
| PM_2.5_ (μg/m^3^) | 78.26±39.44 | 20.4 | 48.07 | 71.14 | 98.21 | 217.71 |
| NO_2_ (μg/m^3^) | 49.31±16.67 | 15.57 | 37.79 | 47.71 | 59.79 | 91.14 |
| SO_2_ (μg/m^3^) | 40.66±23.94 | 6.00 | 23.36 | 37.71 | 55.14 | 124.14 |
| Rizhao |  |  |  |  |  |  |
| Ambient temperature (℃) | 13.28±9.39 | -6.15 | 4.04 | 13.04 | 21.98 | 28.77 |
| Relative humidity (%) | 68.24±11.65 | 36.14 | 59.46 | 69.71 | 78.21 | 89.86 |
| Wind speed (m/s) | 2.42±0.52 | 1.45 | 2.03 | 2.33 | 2.84 | 3.74 |
| Cumulative precipitation (mm) | 11.67±18.86 | 0 | 0.03 | 2.85 | 16.88 | 136.35 |
| PM_2.5_ (μg/m^3^) | 60.82±29.49 | 14.14 | 41.50 | 53.85 | 72.92 | 200.43 |
| NO_2_ (μg/m^3^) | 37.00±11.80 | 11.43 | 28.43 | 37.00 | 44.52 | 73.43 |
| SO_2_ (μg/m^3^) | 24.33±13.35 | 6.29 | 15.36 | 20.14 | 27.79 | 78.29 |

NO_2,_ nitrogen dioxide; *P*_25_, the 25th percentile; *P*_75_, the 75th percentile; PM_2.5_, particulate matter less than 2.5 μm aerodynamic diameter; SD, standard deviation; SO_2,_ sulfur dioxide.

**Supplementary Table 2.** Tests of heterogeneity for second-stage in multivariate meta- analyses.

| Region | Cochran *Q* test | | | *I*² (%) |
| --- | --- | --- | --- | --- |
|  | *Q* | df | *P* |  |
| Central Shandong | 44.93 | 16 | <0.01 | 64.39 |
| Jiaodong Peninsula | 9.23 | 8 | 0.32 | 13.33 |
| North Shandong | 47.75 | 12 | <0.01 | 74.87 |
| South Shandong | 13.59 | 16 | 0.63 | 17.73 |
| Shandong Province | 150.92 | 64 | <0.01 | 57.59 |

df, degree of freedom.


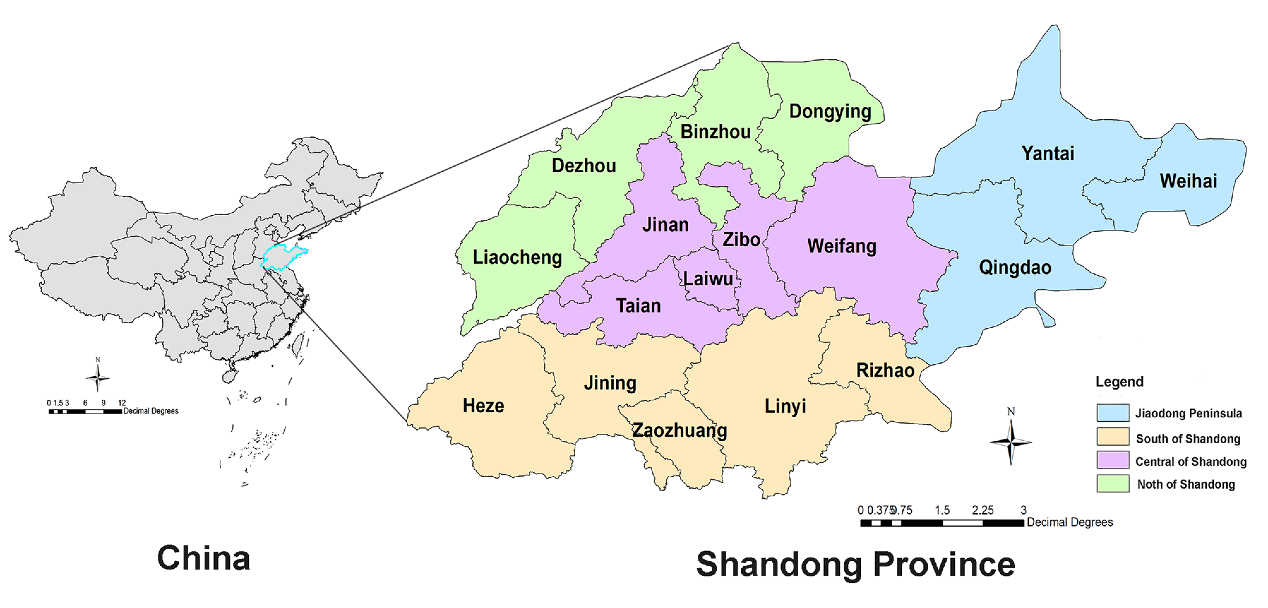


**Supplementary Figure 1.** Location of the study area in China and geographic regions of Shandong Province. The China and Shandong maps were created with ArcGIS software based on the public geographical data downloaded from Resource and Environment Science and Data Center, Institute of Geographic Sciences and Natural Resources Research, CAS (https://www.resdc.cn/).


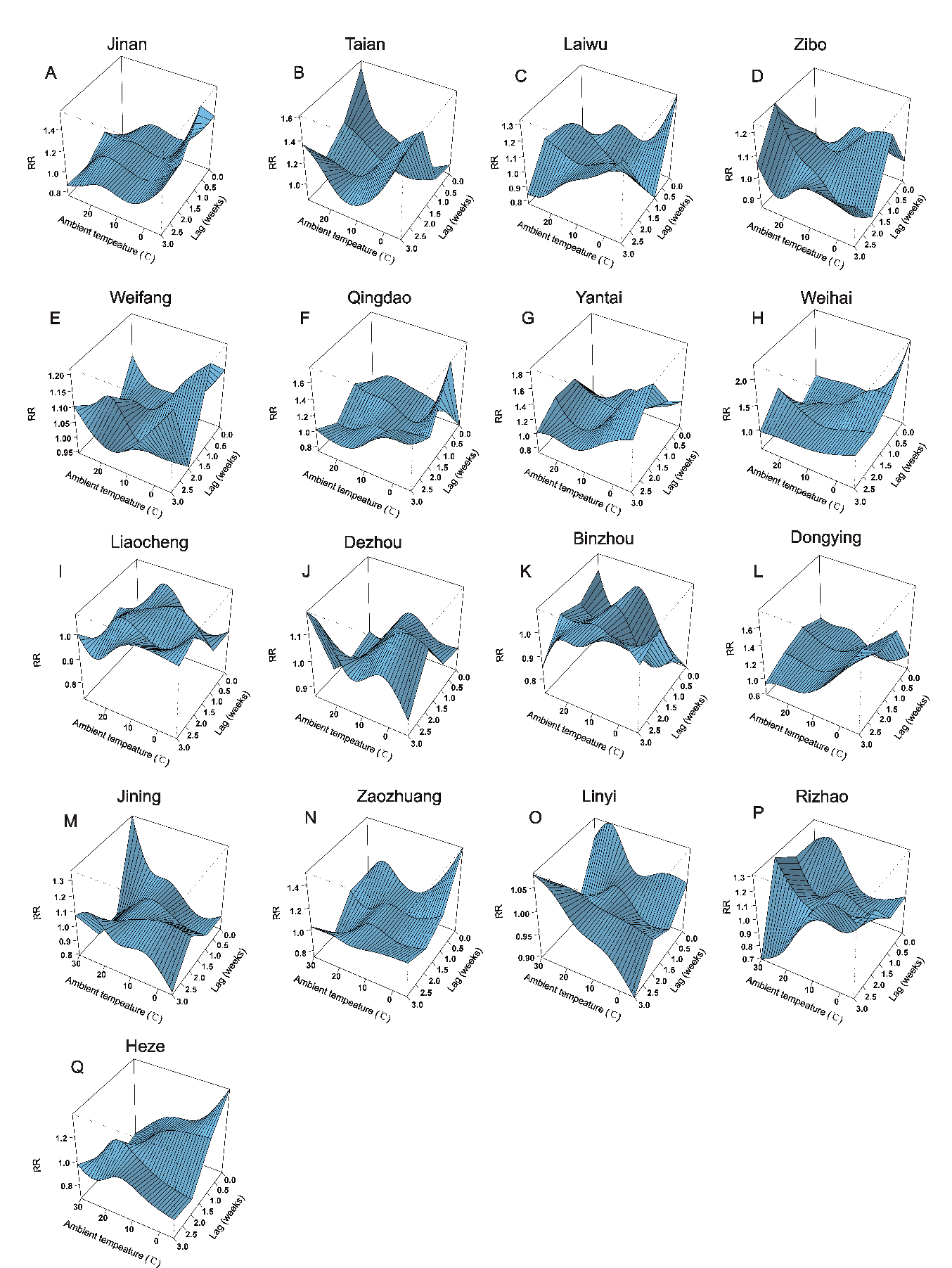


**Supplementary Figure 2.** Three-dimensional (3D) exposure-lag-response plot for weekly mean ambient temperature and ILI in 17 cities. ILI, influenza-like-illness; RR, relative risk.


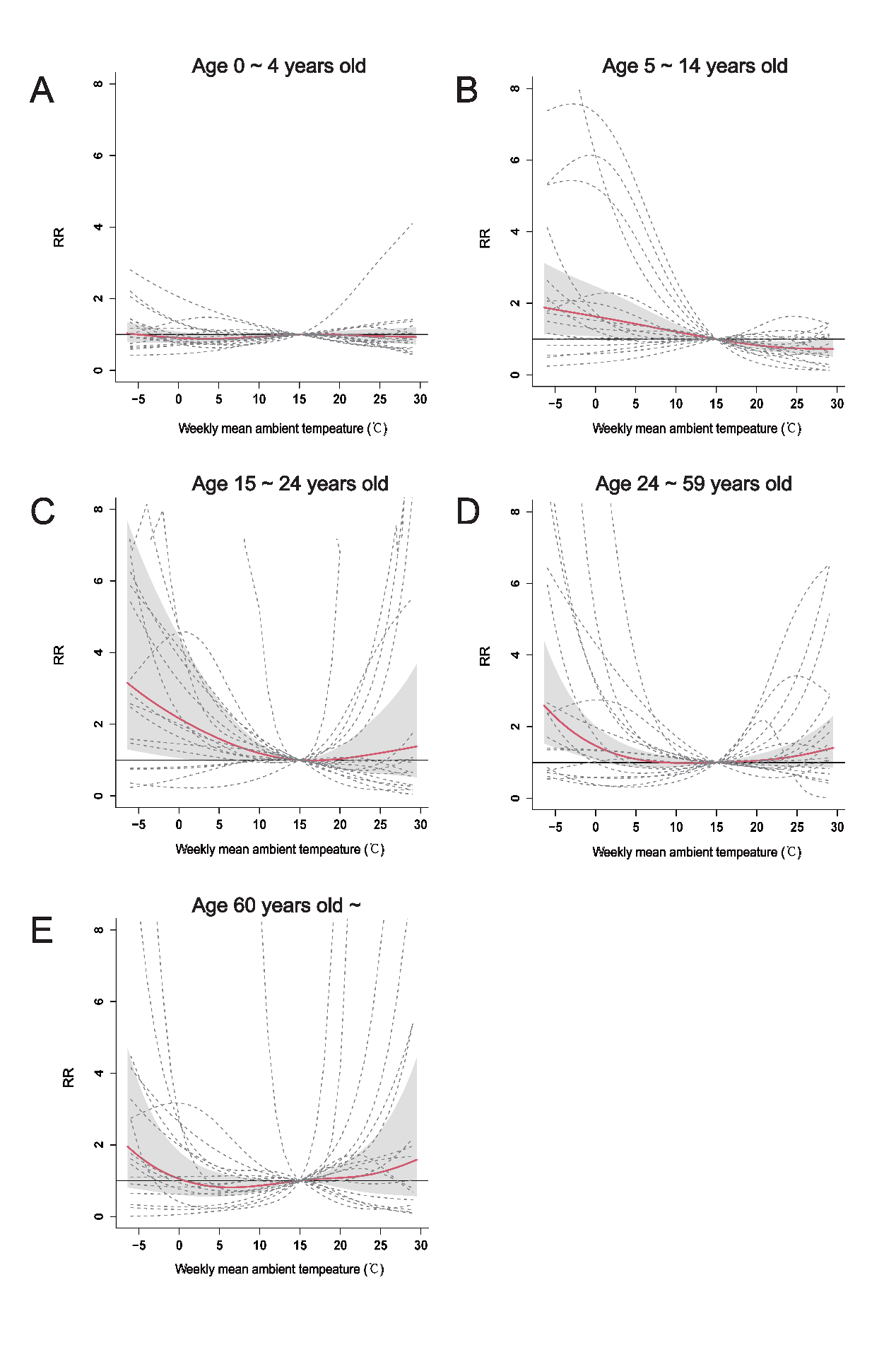


**Supplementary Figure 3.** The overall pooled estimate between weekly mean ambient temperature and ILI in different age groups. ILI, influenza-like-illness; RR, relative risk.


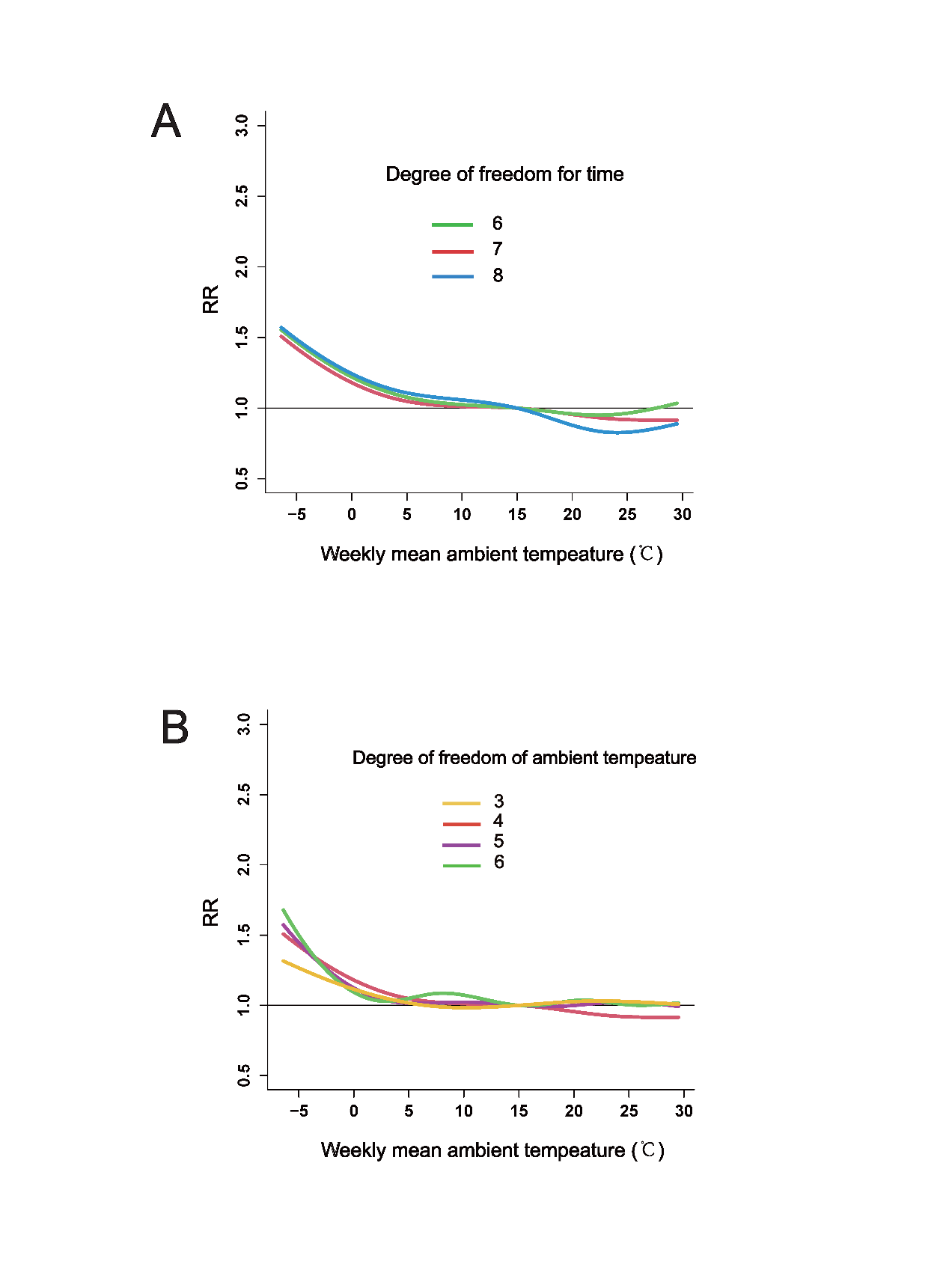


**Supplementary Figure 4.** Pooled estimates of weekly ambient temperature on ILI when changing the df for time (A) and ambient temperature (B). df, degree of freedom; ILI, influenza-like-illness; RR, relative risk.


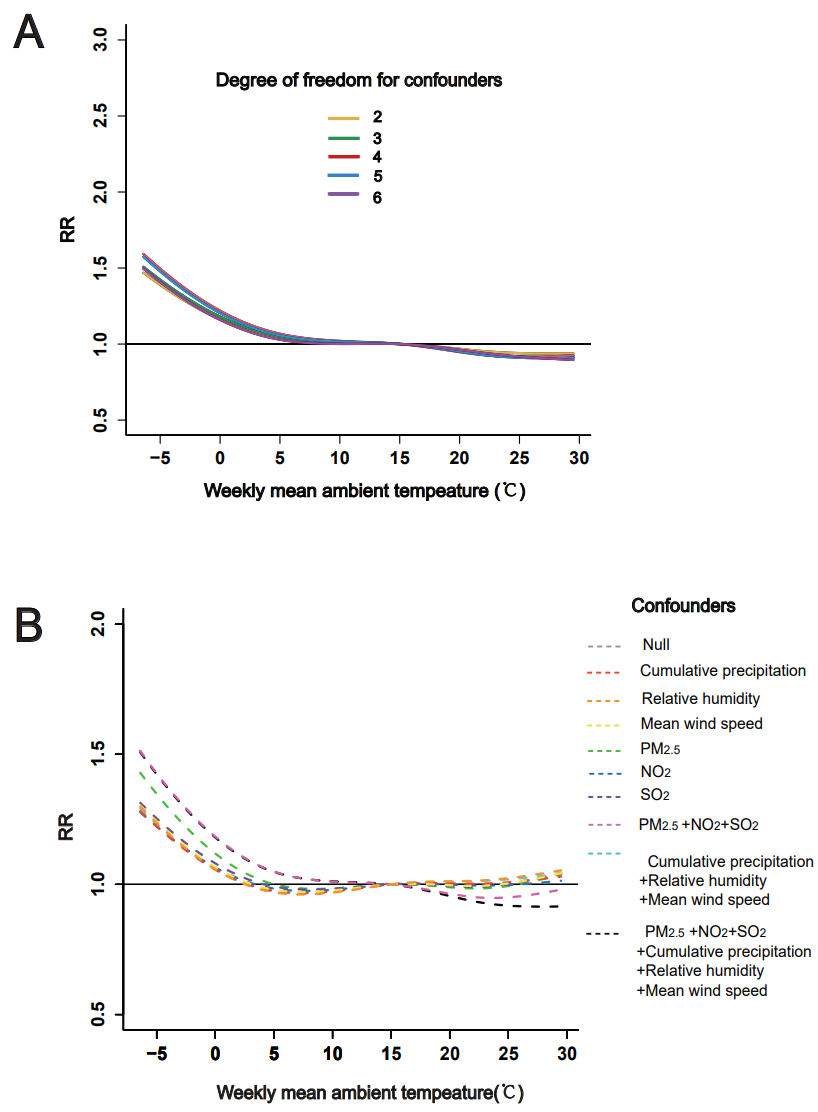


**Supplementary Figure 5.** Pooled estimates of weekly ambient temperature on ILI when changing the df for confounders (A) and adjusting for confounders (B). ILI, influenza-like-illness; NO_2_, nitrogen dioxide; PM_2.5_, particulate matter < 2.5 µm in aerodynamic diameter; RR, relative risk; SO_2_, sulfur dioxide.


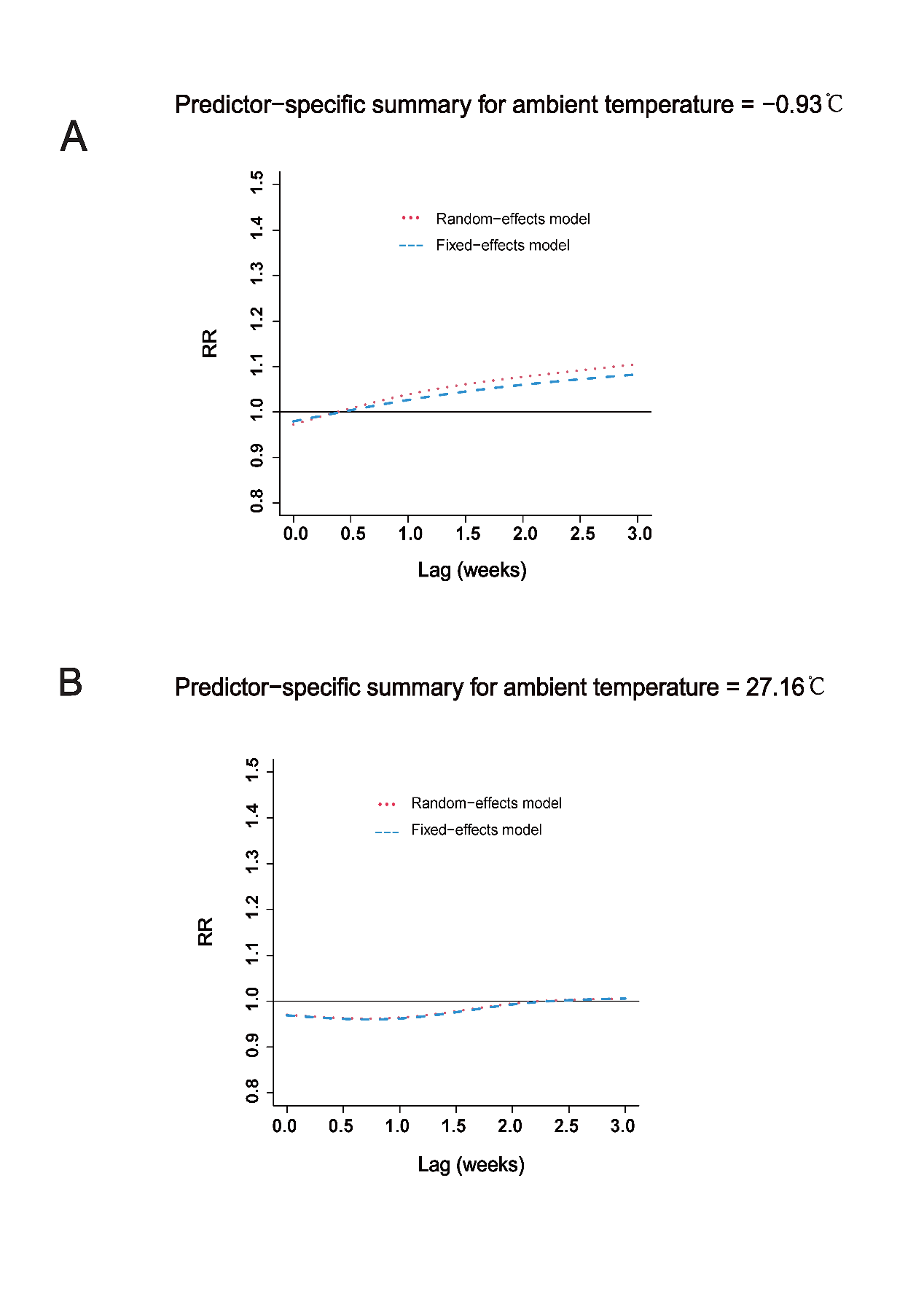


**Supplementary Figure 6.** Pooled estimates of weekly ambient temperature on ILI when changing the different effect models. ILI, influenza-like-illness. (A) cold effect; (B) hot effect.


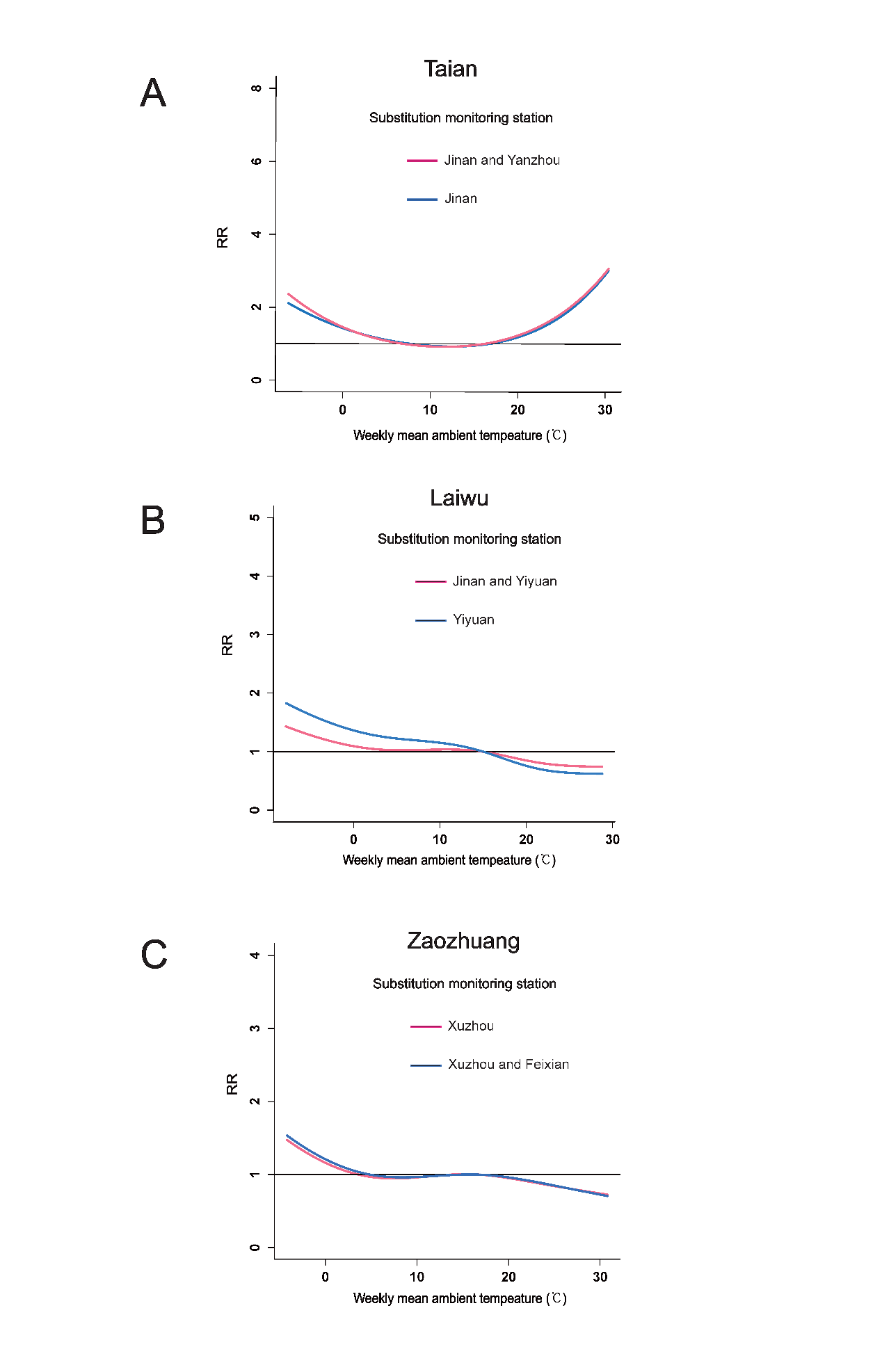


**Supplementary Figure 7.** The city-specific cumulative effects of weekly mean ambient temperature on ILI when changing average metrics and selection of stations to replace missing data. ILI, influenza-like-illness; RR, relative risk. (A) Taian; (B) Laiwu; (C) Zaozhuang.


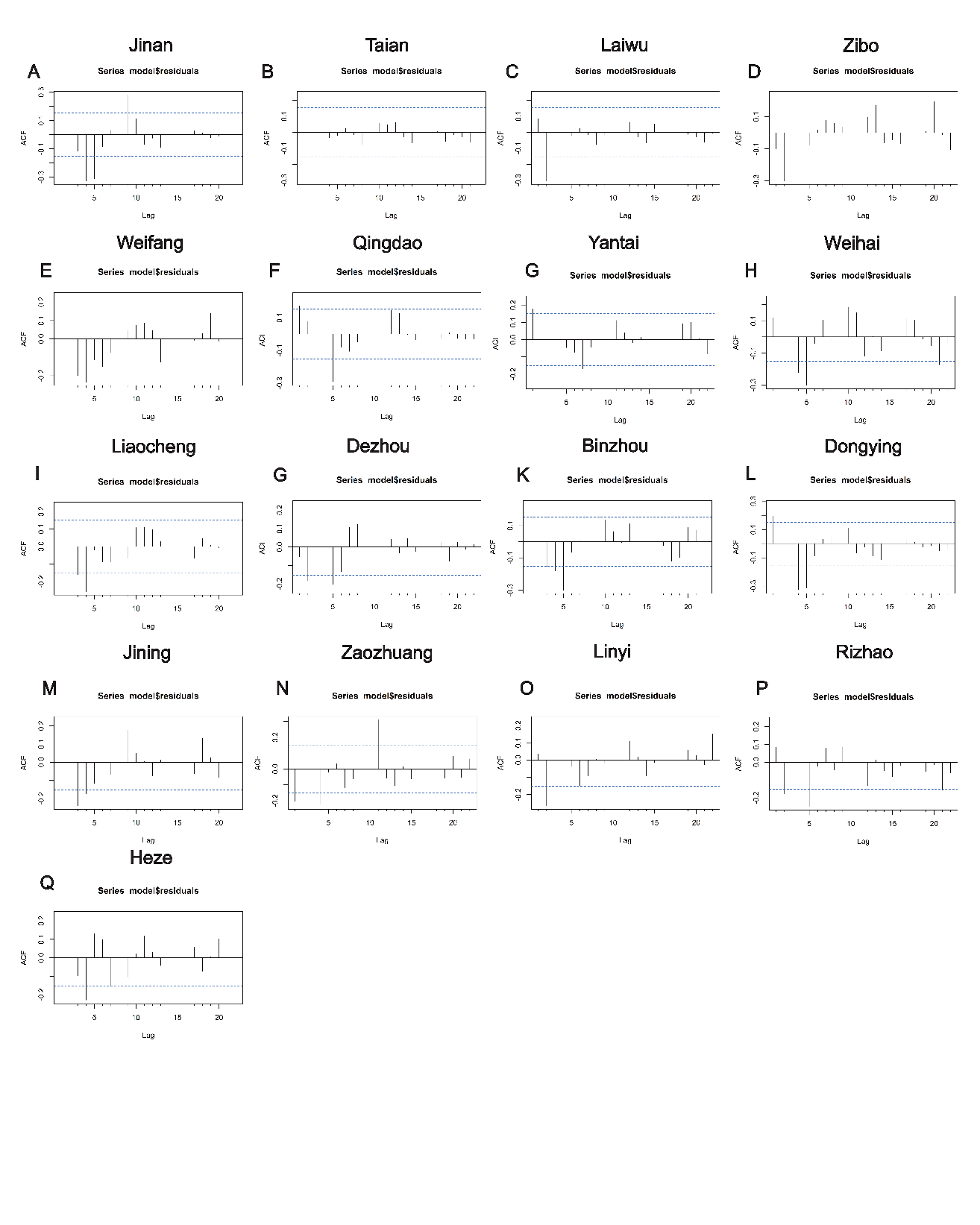


**Supplementary Figure 8.** Autocorrelation function (ACF) plots of residuals in 17 cities derived from the city-specific DLNM models. DLNM, distributed lag non-linear model.


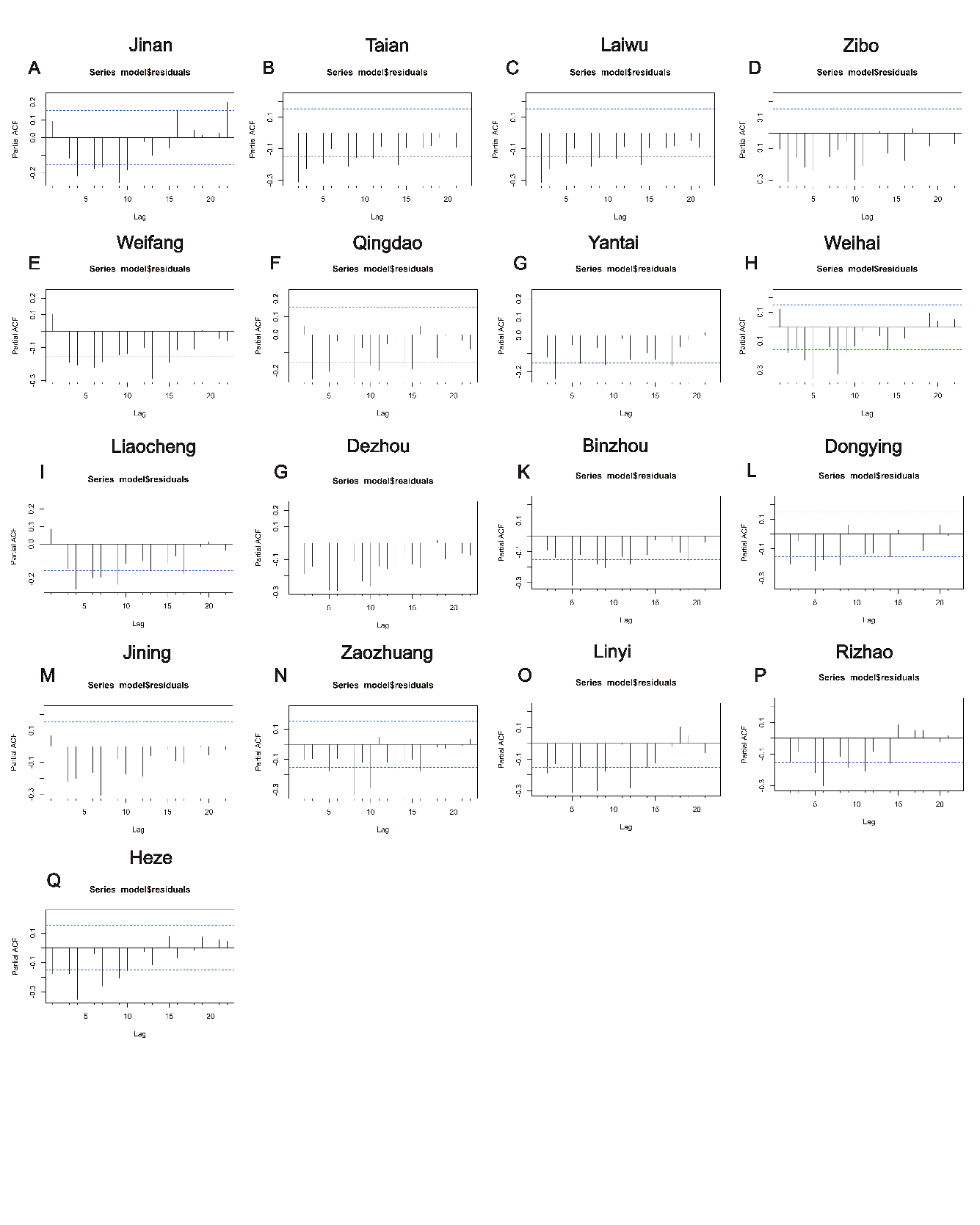


**Supplementary Figure 9.** Partial autocorrelation function (PACF) of residuals in 17 cities derived from the city-specific DLNM models. DLNM, distributed lag non-linear model.


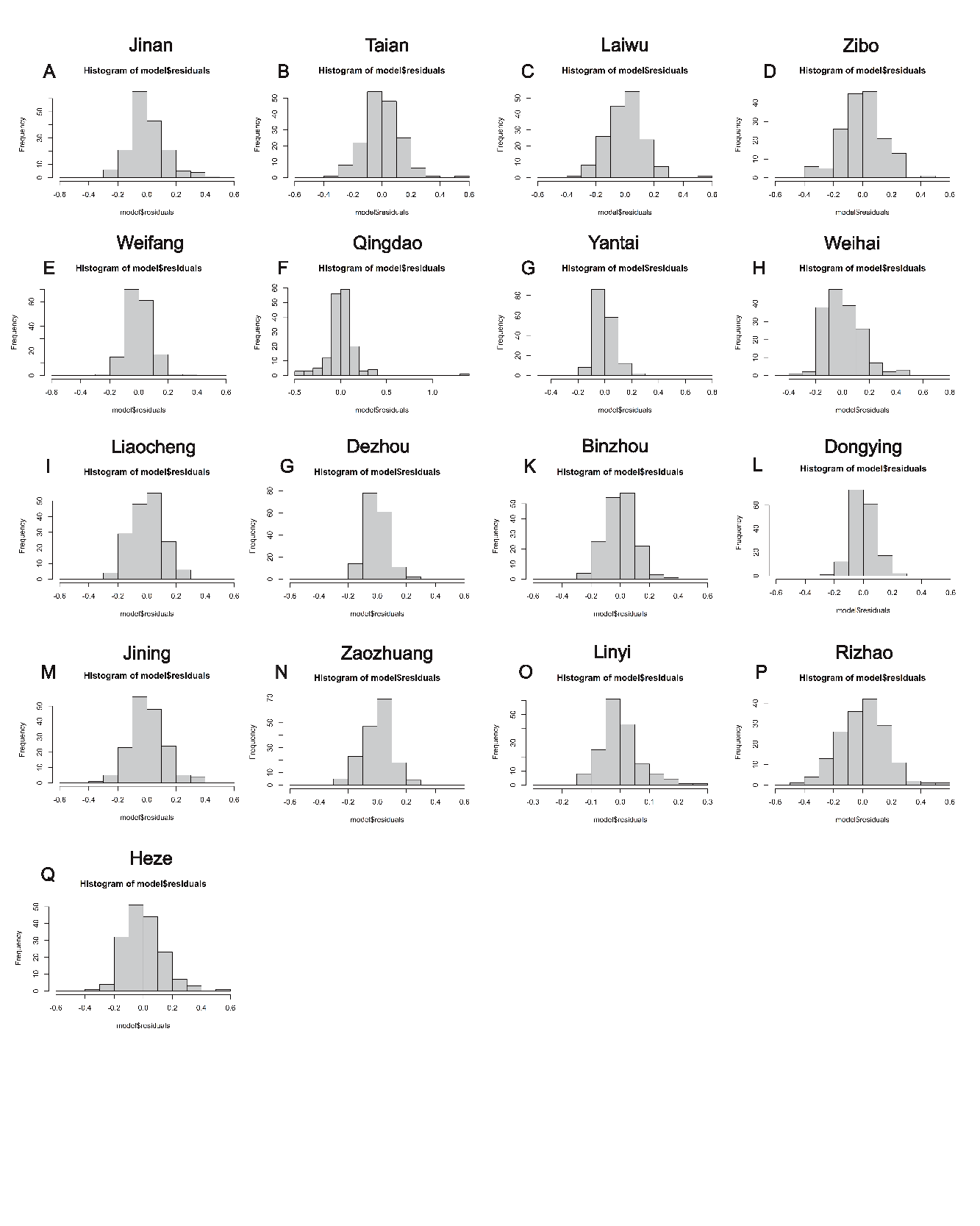
 **Supplementary Figure 10.** Histogram of residuals in 17 cities derived from the city-specific DLNM models. DLNM, distributed lag non-linear model.
